# Immunoproteomic analysis of *Borrelia miyamotoi* for the identification of serodiagnostic antigens

**DOI:** 10.1038/s41598-019-53248-5

**Published:** 2019-11-14

**Authors:** Emma K. Harris, Marisa R. Harton, Maria Angela de Mello Marques, John T. Belisle, Claudia R. Molins, Nicole Breuner, Gary P. Wormser, Robert D. Gilmore

**Affiliations:** 1 Bacterial Diseases Branch, Division of Vector-Borne Diseases, National Center for Emerging and Zoonotic Infectious Diseases, Centers for Disease Control and Prevention, Fort Collins, CO USA; 2 Department of Microbiology, Immunology and Pathology, Colorado State University, Fort Collins, CO USA; 3 Department of Medicine, Division of Infectious Disease, New York Medical College, Valhalla, NY USA

**Keywords:** Infectious-disease diagnostics, Bacteriology

## Abstract

The tick-borne spirochete, *Borrelia miyamotoi*, is an emerging pathogen of public health significance. Current *B*. *miyamotoi* serodiagnostic testing depends on reactivity against GlpQ which is not highly sensitive on acute phase serum samples. Additionally, anti-*B*. *miyamotoi* antibodies can cross-react with C6 antigen testing for *B*. *burgdorferi*, the causative agent of Lyme disease, underscoring the need for improved serological assays that produce accurate diagnostic results. We performed an immunoproteomics analysis of *B*. *miyamotoi* proteins to identify novel serodiagnostic antigens. Sera from mice infected with *B*. *miyamotoi* by subcutaneous inoculation or tick bite were collected for immunoblotting against *B*. *miyamotoi* membrane-associated proteins separated by 2-dimensional electrophoresis (2DE). In total, 88 proteins in 40 2DE immunoreactive spots were identified via mass spectrometry. Multiple variable large proteins (Vlps) and a putative lipoprotein were among those identified and analyzed. Reactivity of anti-*B*. *miyamotoi* sera against recombinant Vlps and the putative lipoprotein confirmed their immunogenicity. Mouse anti-*B*. *burgdorferi* serum was cross-reactive to all recombinant Vlps, but not against the putative lipoprotein by IgG. Furthermore, antibodies against the recombinant putative lipoprotein were present in serum from a *B*. *miyamotoi*-infected human patient, but not a Lyme disease patient. Results presented here provide a comprehensive profile of *B*. *miyamotoi* antigens that induce the host immune response and identify a putative lipoprotein as a potentially specific antigen for *B*. *miyamotoi* serodetection.

## Introduction

The tick-borne spirochete *Borrelia miyamotoi* is an emergent pathogen of worldwide public health importance. Despite its phylogenetic grouping with relapsing fever *Borrelia* (RFB), which are transmitted by argasid (or soft) ticks, *B*. *miyamotoi* is transmitted by ixodid (or hard) tick species. In North America, vectors include *Ixodes scapularis* and *I*. *pacificus*, which are also hosts for the causative agent of Lyme disease (LD), *Borrelia burgdorferi*. Prevalence in ticks ranges from 0.02 to 10% in *I*. *scapularis* and *Ixodes pacificus* populations in the United States^1–4^. Though the true burden of *B.miyamotoi* disease (BMD) on human disease is still being realized, current research has estimated human prevalence to be approximately 0.84–17% among individuals parasitized by ticks^1,3,5–8^. Symptoms associated with BMD are often non-specific, complicating diagnosis in endemic locations with high LD incidence^9,10^. Furthermore, there remains a relative paucity of information regarding immunogenic characterization for *B*. *miyamotoi* infection in vertebrate hosts.

To date, sensitive and specific serology-based diagnostics for BMD are experimental, lacking in commercial validation and production. Laboratory serodiagnostics target the *B*. *miyamotoi* antigen glycerophosphodiester phosphodiesterase (GlpQ), a gene product present in RFB but absent in Lyme *Borrelia*. However, weak antibody response against GlpQ has been reported in a number of confirmed BMD cases, particularly acute phase serum samples^5,11,12^. Recent studies have suggested that serodiagnostic sensitivity for BMD can be improved by assaying GlpQ reactivity in conjunction with variable major proteins (Vmps) (i.e. variable large and small proteins)^13,14^. Vmps are responsible for the multiphasic antigenic variation that produces fever relapses characteristic of RFB infections^15^. However, antibodies produced against Vmps may be susceptible to cross-reactivity with the orthologous *B*. *burgdorferi* protein Vmp-like sequence expressed (VlsE), which comprises the sensitive C6 antigen used for LD testing^16,17^. A recent retrospective study highlighted that serum from 22 of 24 (91.7%) BMD-positive patients possessed cross-reactive antibodies to the C6 antigen^12^. Therefore, we hypothesized that a comprehensive immunoproteomic analysis of the host antibody response against *B*. *miyamotoi* infection would reveal unique antigens to augment GlpQ and Vmps for improved serodiagnostic detection of BMD.

In this study, antiserum from *B*. *miyamotoi*-infected mice applied to immunoblots of *B*. *miyamotoi* proteins separated by 2-dimensional electrophoresis (2DE) defined the immunogenic protein profile of this pathogen. Mass spectrometry was used to identify putative serum-reactive proteins leading to the identification of a novel lipoprotein antigen with the potential to differentiate BMD from LD. These analyses demonstrate considerable variability in the antibody response between mice infected via subcutaneous inoculation and tick bite, as well as across the course of infection.

## Results

### *B*. *miyamotoi*-infected murine hosts elicit an immune response against multiple proteins regardless of inoculation route

**Figure 1:**
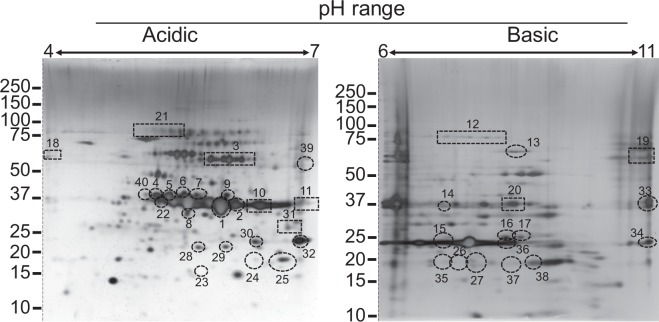
Silver stain representation of *B*. *miyamotoi* LB-2001 membrane-associated proteins. A defined spot profile was established by using approximately 100 µg of protein applied to IPG strips for IEF across pH 4–7 and 6–11. Second dimension 4–12% SDS-PAGE separated proteins by molecular weight (denoted by kDa on left of panels). Immunoreactive protein spots against antibodies present in *B*. *miyamotoi*-infected murine hosts are circled and numbered with corresponding identities listed in Table 1 and Supplemental Table 1. Dashed lines demarcate where the gel has been cropped to exclude molecular weight markers. Uncropped gel images can be viewed in Supplemental Fig. 2.

*B*. *miyamotoi* membrane-associated proteins were purified, fractionated by 2DE (Fig. 1), and subjected to immunoblotting with antiserum from mice infected by either needle inoculation or tick bite and sacrificed at 8 or 40 days. Proteins corresponding to the immunoreactive spots from the blots were identified by mass spectrometry. Protein identifications for each spot noted below are listed in Table 1 and corresponding raw data are located in Supplemental Table 1.

**Table 1:** Protein identities corresponding to immunoreactive spots.

GO term^a^	Spot no.	Protein ID	pI^b^	NCBI accession ID	Pred MW in kDa^c^	No. of unique peptides
**CC**^**d**^**: Membrane**						
	1, 3, 10, 11, 22, 31, 32, 39, 40	VlpC2^g^	5.84	ALU64348.1	35	26, 5, 28, 24, 14, 5, 9, 4, 4
	2	Vlp5^g^	5.73	AOW96300.1	35	2
	2, 4, 5, 6, 7, 22, 33	VlpD2^g^	5.55	ALM31567.1	34*	3, 5, 9, 14, 5, 12, 4
	4, 5, 6, 7, 9, 22, 33	VlpD8^g^	5.43	ALU64349.1	33*	29, 32, 22, 7, 5, 16, 7
	4, 5, 6, 7, 9, 10, 22	VlpD9^g^	5.96	ALU64350.1	34*	2, 3, 5, 9, 18, 2, 2
	4, 5, 6, 7, 22	Vlp15/16^g^	5.61	AOW96282.1	33*	5, 7, 7, 5, 3
	4, 5, 6, 22	VlpD1^g^	5.62	ALM31565.1	34	4, 8, 11, 8
	5, 6, 7, 9, 22	VlpD10^g^	5.77	ALU64352.1	33*	2, 3, 15, 4, 2
	14, 16, 20, 33, 38	Vlp^g^	5.87	AOW96324.1	33*	10, 4, 7, 14, 2
	22	Vlp^g^	5.89	AOW96302.1	34	5
	6, 10, 11, 15, 16, 17, 20 25, 26, 27, 30, 31, 32, 34, 35, 36	Vsp1^h^	6.18	AJA67245.2	22	3, 3, 5, 8, 4, 4, 2, 11, 3, 4, 20, 5, 20, 6, 2, 11
	11	HflK	5.79	AGT27198.1	35	8
	11	HflC	9.52	AGT27199.1	37	8
	23	DUF1640 domain-containing protein	5.42	AOW96294.1	18	3
	18	Ribonuclease Y	7.65	AGT27464.1	58	3
**CC**^**d**^**: flagellum associated**						
	1, 2, 4, 5, 6, 7, 8, 9, 10, 11, 21, 22, 30, 32, 33	Flagellin	5.36	AGT27144.1	35	11, 13, 9, 10, 12, 11, 12, 10, 11, 10, 10, 13, 11, 10, 12
	6, 22, 33	Flagellar assembly protein-FliH	5.28	AGT27286.1	35	14, 3, 3
	28	Flagellar protein	5.33	AGT27283.1	24	2
	2, 11, 14	Flagellar protein	8.38	AGT27611.1	39	10, 2, 3
	23	Flagellar hook assembly protein-FlgD	5.49	AGT27281.1	16	2
	38	Flagellar basal-body rod protein-FlgC	8.92	AGT27290.1	17	2
	40	Flagellar motor switch protein-FliM	5.09	AGT27275.1	39	2
**MF**^**e**^**: phosphoric diester hydrolase activity**						
	1, 2, 5, 6, 8, 11, 22	GlpQ^i^	5.68	AGT27237.1	39	16, 10, 2, 13, 9, 11, 15
**MF**^**e**^**: ATPase and ATP binding**						
	1, 31	Phosphate ABC transporter substrate-binding protein	6.92	AGT27210.1	31	2, 13
	2, 4, 5, 7, 8, 19, 22	60 kDa chaperonin	5.28	AGT27591.1	59	5, 10, 4, 2, 4, 3, 8
	3, 18	ABC transporter substrate binding protein	6.11	AGT27324.1	61	32, 19
	3, 18, 19	ABC transporter substrate binding protein	5.90	AGT27323.1	61	18, 12, 4
	8, 21	Chaperone DnaK	5.00	AGT27477.1	69	7, 40
	10, 11	ATP-dependent zinc metalloprotease-FtsH	8.61	AGT27731.1	71	2, 2
	11	Holliday junction ATP-dependent DNA helicase-RuvB	7.04	AGT27027.1	38	2
	11	ATPase	6.39	AGT27171.1	37	17
	11, 20, 22	ABC-transporter binding protein	9.26	AGT27698.1	35	11, 2, 2
	28, 29	V-type protein ATPase subunit E	5.49	AGT27099.1	23	10, 10
	31	Phosphate import ATP-binding protein-PstB	6.22	AGT27213.1	29	9
	39	ATP-dependent Clp protease ATP-binding subunit-ClpX	7.97	AGT27557.1	48	2
**MF**^**e**^**: protein binding**						
	8, 31	Uncharacterized protein	8.33	WP_082002168.1	30	2, 4
**MF**^**e**^**: catalytic activity**						
	31	Triosephosphate isomerase-TpiA	6.23	AGT27058.1	28	13
	32	5’-methylthioadenosine/S-adenoslyhomocystein nucleosidase	5.97	WP_082583095.1	25	5
	39	Pyruvate kinase	6.18	AGT27339.1	53	21
	39	Inosine-5’-monophosphate dehydrogenase-GuaB	8.48	AJA67229.1	53	7
**MF**^**e**^**: double-stranded RNA binding**						
	11	Threonylcarbamoyl-AMP synthase	9.35	AGT27679.1	38	4
**MF**^**e**^**: translation associated**						
	15, 30, 32	50 S ribosomal protein-L25	6.77	AGT27728.1	21	3, 2, 6
	18	Lysine-tRNA ligase	8.90	AGT27602.1	61	8
	23, 25	30 S ribosomal protein-S10	10.05	AGT27452.1	12	2, 2
	24	Elongation factor G	5.50	AGT27495.1	77	2
	25	50 S ribosomal protein L21	9.70	AGT27721.1	12	4
	26, 27	50 S ribosomal protein L5	9.60	AGT26990.1	20	2, 4
	26, 27, 35	50 S ribosomal protein L9	9.62	AGT27115.1	19	4, 4, 2
	32	50 S ribosomal protein L3	9.83	AGT26984.1	23	2
	37	50 S ribosomal protein L19	10.23	AGT27642.1	17	2
	38	30 S ribosomal protein S7	9.99	AGT26980.1	18	2
**MF**^**e**^**: GTP-binding**						
	7	Ribosome-binding ATPase YchF	5.40	AGT27227.1	41	3
**BP**^**f**^**: metabolic processes**						
	2	Ornithine carbamoyltransferase	5.74	WP_043867909.1	37	7
	2	Phosphoglycerate kinase	5.84	AGT27059.1	43	4
	4, 5, 22	Enolase	5.05	AGT27330.1	47	3, 2, 2
	11, 14, 20, 33	GAPDH^j^	8.32	AGT27060.1	36	17, 3, 16, 2
**BP**^**f**^**: cell morphogenesis**						
	11	Rod shape-determining protein	6.86	AGT27659.1	38	17
**BP**^**f**^**: proteolysis**						
	10	Serine protease	8.99	WP_082002164.1	53	3
	39	M18 family aminopeptidase	6.03	AGT27354.1	52	7
**BP**^**f**^**: transcription associated**						
	18	Transcription termination/antitermination protein-NusA	4.74	AGT27741.1	55	2
	4, 5, 6, 22, 33	DNA-directed RNA polymerase subunit alpha-RpoA	5.11	AGT27462.1	38	17, 16, 15, 18, 3
	28, 29	Transcription termination/antitermination protein-NusG	5.48	AGT27376.1	21	12, 6
**BP**^**f**^**: polyamine binding**						
	11	Spermidine/putrescine import ATP-binding protein	8.78	AGT27580.1	40	3
	22	Sperimidine/putrescine ABC transporter substrate-binding protein	5.46	AGT27583.1	41	13
**BP**^**f**^**: protein folding**						
	32	Protein GrpE	5.38	AGT27478.1	21	4
**BP**^**f**^**: chemotaxis**						
	37, 38	Probable chemoreceptor glutamine deamidase-CheD	8.63	AGT27553.1	18	3, 4
**Unassigned**						
	4, 8, 12, 37	Uncharacterized protein	8.22	AGT27688.1	77	4, 2, 13, 2
	6	Uncharacterized protein	5.63	AOW96351.1	35	6
	8	Uncharacterized protein	5.18	AGT27657.1	30	5
	11, 20	Uncharacterized protein	9.02	AGT27150.2	44	3, 4
	11	Uncharacterized protein	6.15	AOW96199.1	37	2
	11, 20	Uncharacterized protein	8.91	AGT27497.1	42	2, 2
	13	Uncharacterized protein	8.80	AGT27550.1	67*	16
	21	Uncharacterized protein	5.09	AOW96138.1	28	3
	22	Uncharacterized protein	5.15	AOW96267.1	35	2
	23	Uncharacterized protein	5.44	AGT27223.1	15	5
	23	Uncharacterized protein	5.72	AOW96390.1	19	5
	25	Uncharacterized protein	5.91	AOW96369.1	29	3
	23, 24, 25	Uncharacterized protein	6.18	AOW96394.1	15*	8, 10, 6
	29	Uncharacterized protein	8.37	AGT27607.1	25	5
	30	Uncharacterized protein	6.86	AGT27219.1	28	4
	32	Uncharacterized protein	7.66	AGT27434.1	27	2
	35	Uncharacterized protein	6.21	AGT27719.1	18*	3
	39	Uncharacterized protein	8.11	AGT27044.1	59	3
	23	Heat-shock protein	5.72	WP_043867868.1	17	8
	11	Carboxylesterase	9.53	AGT27588.1	38	2
	16, 30, 36	Peptidoglycan-binding protein-LysM	8.52	AGT27318.1	44*	5, 2, 2,
	4, 5, 6, 8, 11, 22	Putative lipoprotein	5.24	ALN43426.1	35	2, 3, 4, 3, 3, 13

Proteins are categorized according to common gene ontology (GO) terms as listed in UniProtKB. Each spot number (no.) corresponds respectively to the no. of unique peptides detected during protein identification.

^*a*^GO terms = gene ontology terms; ^*b*^pI = isoelectric point; ^*c*^Pred MW = predicted molecular weight; ^*d*^CC = cellular component; ^*e*^MF = molecular function; ^*f*^BP = biological process; ^*g*^Vlp = variable large protein; ^*h*^Vsp = Variable small protein; ^*i*^GlpQ = glycerophosphodiester phosphodiesterase; ^*j*^GAPDH = glyceraldehyde-3-phosphate dehydrogenase; * = predicted pI and MW excludes signal sequence if detected via ExPASy.

**Figure 2:**
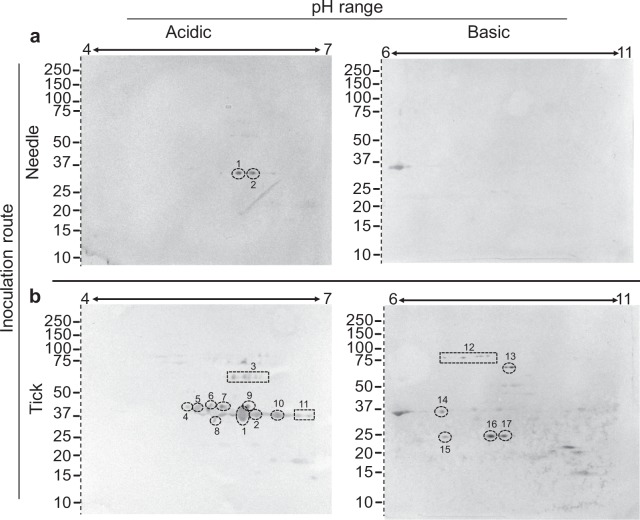
IgM 2DE immunoblot with sera from *B*. *miyamotoi*-infected CD1 mice collected at 8 dpi. Antigen recognition was examined in response to needle inoculation of *B*. *miyamotoi* LB-2001 (**a**) or through tick bite from *B*. *miyamotoi*-*i*nfected *I*. *scapularis* originating from Minnesota (**b**). For both sets of immunoblots an acidic (pH 4–7) and basic (6–11) pH range were utilized. Sera were diluted 1:200 for immunoblotting. Immunogenic proteins spots (number 1–17) were excised from a corresponding silver stain 2DE and identified by mass spectrometry. All proteins were consistently numbered across immunoblot and time point and resulting identities from individual spots listed in Table 1 and Supplemental Table 1. Molecular weight of proteins are expressed in kDa. Dashed lines demarcate where the gel has been cropped to exclude molecular weight markers. Uncropped blots can be viewed in Supplemental Fig. 2.

Acute IgM antibody response from needle inoculated mice at 8 days post-inoculation (dpi) resulted in two reactive spots constituting 10 protein identities: VlpC2, Vlp5, VlpD2, flagellin, a flagellar protein, GlpQ, phosphate ABC transporter substrate-binding protein, 60 kDa chaperonin, ornithine carbamoyltransferase, and phosphoglycerate kinase (Fig. 2a; Table 1). IgM immunoblotting with serum collected at 8 dpi derived from tick bite inoculation reacted with 17 immunogenic spots representing 47 protein identities (Fig. 2b; Table 1). This included immunoreactive spots number 1 and 2 which were recognized by serum at 8 dpi across both needle and tick bite inoculation routes. Identification of additional proteins recognized by mouse serum from 8 dpi via tick bite included proteins assigned the following gene ontology associations: membrane (VlpD8, VlpD9, Vlp 15/16, VlpD1, VlpD10, Vlp, and Vsp1); flagellum associated; ATPase and ATP-binding; protein binding; double-stranded RNA binding; translation associated; GTP-binding; metabolic processes; cell morphogenesis; proteolysis; transcription associated; polyamine binding; and unassigned (including uncharacterized proteins and a putative lipoprotein) (Fig. 2b; Table 1).

**Figure 3:**
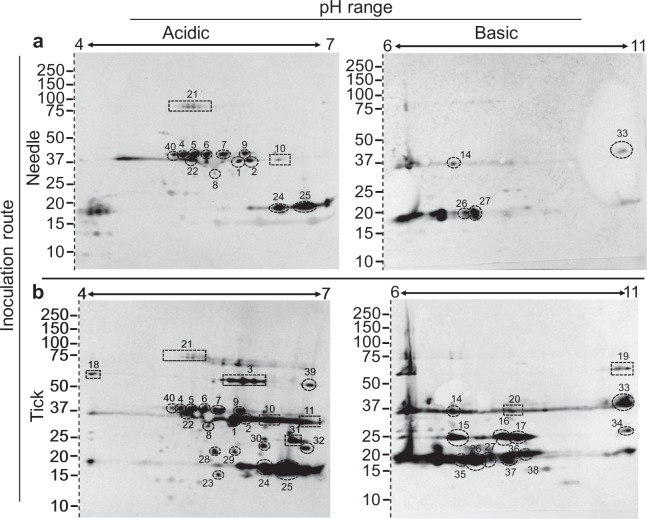
IgG 2DE immunoblot with sera from *B*. *miyamotoi*-infected CD1 mice collected at 40 dpi. Antigen recognition was examined in response to needle inoculation of *B*. *miyamotoi* LB-2001 (**a**) or through tick bite from *B*. *miyamotoi*-infected *I*. *scapularis* originating from Minnesota (**b**). For both sets of immunoblots an acidic (pH 4–7) and basic (6–11) pH range were utilized. Sera were diluted 1:200 for immunoblotting. Immunogenic protein spots (numbers 1–11 and 14–40) were excised from a corresponding silver stain 2DE and identified by mass spectrometry. All proteins were consistently numbered across immunoblot and time point and resulting identities from individual spots listed in Table 1 and Supplemental Table 1. Molecular weight of proteins are expressed in kDa. Dashed lines demarcate where the gel has been cropped to exclude molecular weight markers. Uncropped blots can be viewed in Supplemental Fig. 2.

The largest number of immunoreactive spots was observed with sera collected at 40 dpi (IgG). Spots 1–11 and 14–17, originally detected at 8 dpi, were again observed at this time point including nine Vlps and Vsp1 (Fig. 3). Twenty-three spots were uniquely observed with sera from 40 dpi. Eight of the 23 unique spots (21, 22, 24, 25, 26, 27, 33, and 40) were detected across immunoblots probed with both needle and tick bite inoculated mouse serum of which protein identities were assigned the following gene ontology associations: membrane (multiple Vlps and Vsp1); flagellum associated; phosphoric diester hydrolase activity (GlpQ); ATPase and ATP-binding; translation associated; metabolic processes; transcription associated; polyamine binding; and unassigned (including the previously identified putative lipoprotein) (Fig. 3a,b; Table 1). The remaining 15 immunoreactive spots recognized only in serum from tick bite inoculated mice represented 47 individual proteins, 18 of which had been identified within other spots across previous data points (i.e. 8 dpi needle or tick inoculated and 40 dpi needle inoculated). Twenty-nine proteins were uniquely identified in serum collected 40 days post-tick inoculation and assigned the following gene ontology associations: flagellum associated; ATPase and ATP-binding; catalytic activity; translation associated; proteolysis; transcription associated; protein folding; chemotaxis; and unassigned (Fig. 3b; Table 1).

### Immunoreactivity against recombinant *B*. *miyamotoi* Vmps and putative lipoprotein validates antigen identity

**Figure 4:**
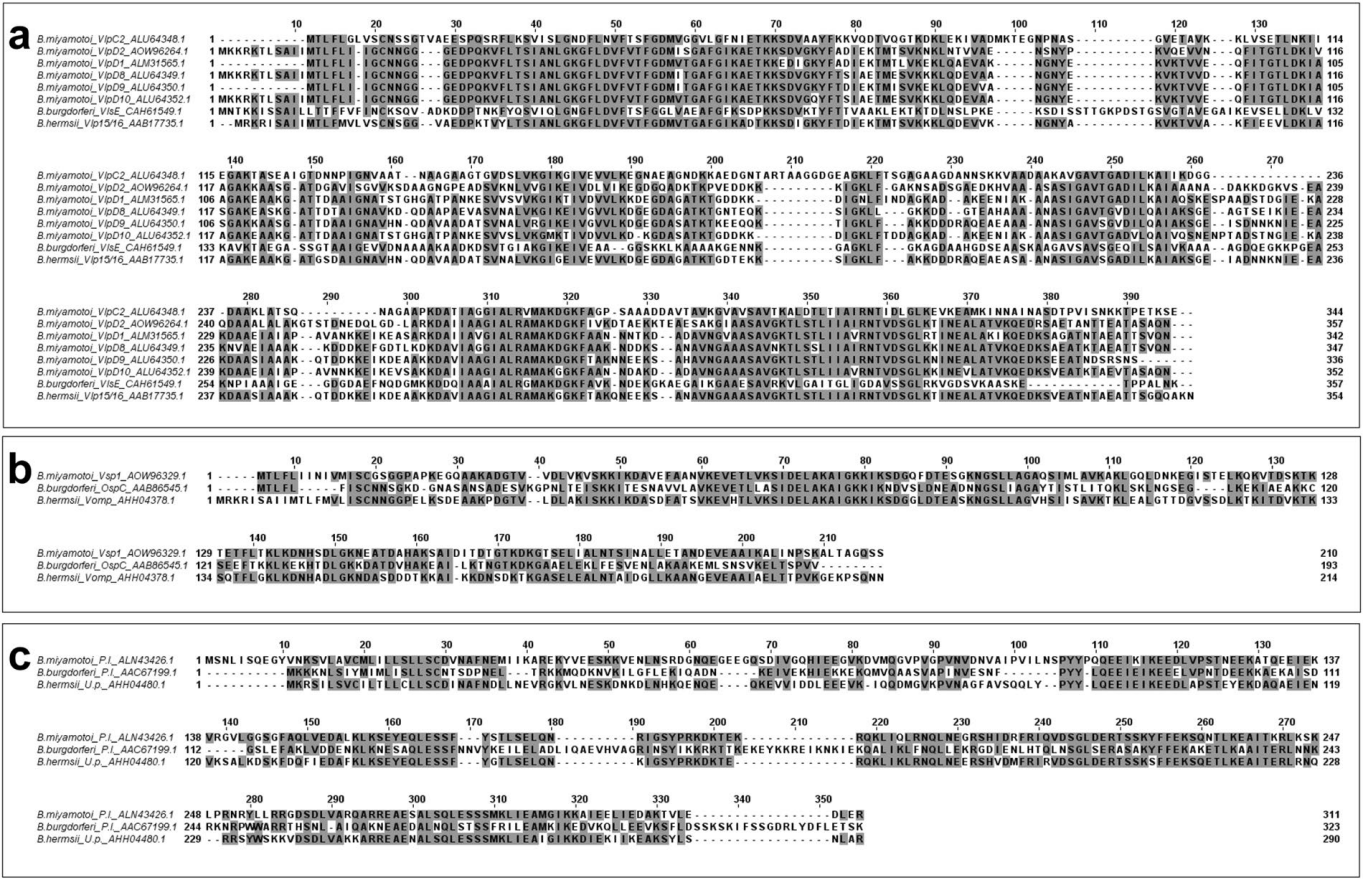
Multiple sequence alignment of *B*. *miyamotoi* proteins identified via mass spectrometry against homologous *B*. *burgdorferi* and *B*. *hermsii* proteins. (**a**) *B*. *miyamotoi* VlpC2, D2, D1, D8, D9, and D10 (NCBI Accession ID: ALU64348.1, AOW96264.1, ALM31565.1, ALU64349.1, ALU64350.1, ALU64352.1) against *B*. *burgdorferi* VlsE (CAH61549.1) and *B*. *hermsii* Vlp 15/16 (AAB17735.1); (**b**) *B*. *miyamotoi* Vsp1 (AOW96329.1) against *B*. *burgdorferi* OspC (AAB86545.1), and *B*. *hermsii* Vomp (variable outer membrane protein) (AHH04378.1); (**c**) *Borrelia miyamotoi* P.l. (putative lipoprotein) (ALN43426.1) against *B*. *burgdorferi* P.l. (AAC67199.1) and *B*. *hermsii* U.p. (uncharacterized protein) (AHH04480.1). Proteins were aligned using MUSCLE and results viewed with Jalview. Gray highlighted regions indicate areas with 60% matching amino acids across compared sequences.

We chose a subset of 10 antigens identified through 2DE immunoblotting to express as recombinant proteins (Table 2; Supplemental Fig. 1a,b) to validate immunoreactivity against antisera used in discovery 2DE immunoblots. Proteins identified by mass spectrometry revealed an uncharacterized protein (AOW96394.1), putative lipoprotein (ALN43426.1), GlpQ, Vsp1, VlpC2, VlpD2, VlpD1, VlpD8, VlpD9, and VlpD10 as potentially antigenic proteins with serodiagnostic utility (Table 2). The amino acid sequence of each *B*. *miyamotoi* protein was aligned with corresponding amino acid sequences from *B*. *burgdorferi* and *B*. *hermsii* proteins with the highest percent identity as reported by the BLASTp database (Fig. 4). All six Vlps were grouped together and aligned against a representative *B*. *burgdorferi* and *B*. *hermsii* protein (i.e. VlsE and Vlp15/16, respectively) which demonstrated the highest amino acid percent identity to any one *B*. *miyamotoi* Vlp. Amino acid alignment comparisons between *B*. *miyamotoi* Vlps and *B*. *burgdorferi* VlsE (Accession CAH61549.1) and *B*. *hermsii* Vlp 15/16 (Accession AAB17735.1) revealed that the six *B*. *miyamotoi* Vlps displayed 36–39% amino acid sequence identity with the *B*. *burgdorferi* VlsE protein and were approximately 76–84% identical to various *B*. *hermsii* Vlps (Fig. 4a; Table 2). Vsp1 shared 46% amino acid identity to *B*. *burgdorferi* OspC (Accession AAB86545.1) and 62% to a *B*. *hermsii* variable outer membrane protein (Accession AHH04378.1) (Fig. 4b; Table 2). The *B*. *miyamotoi* putative lipoprotein amino acid sequence was 39% identical to a *B*. *burgdorferi* putative lipoprotein (Accession AAC67199.1) and 62% to an uncharacterized *B*. *hermsii* protein (Accession AHH04480.1) (Fig. 4c; Table 2). Analysis of the uncharacterized protein demonstrated the lowest percent homology of all selected proteins with 23% identity to *B*. *burgdorferi* OspC (Accession ABQ42952.1) and 50% identity to *B*. *hermsii* arthropod associated lipoprotein (Alp) (Accession GU784814.1) (Table 2)^18^. GlpQ had no *B*. *burgdorferi* orthologue but was 87% identical to *B*. *hermsii* GlpQ (Table 2).

**Table 2:** Recombinant *B*. *miyamotoi* candidate proteins IDs with associated percent identity to *B*. *burgdorferi* and *B*. *hermsii*.

Protein ID	NCBI accession ID	Predicted MW in kDa	*B*. *burgdorferi* % ID (nearest protein-accession ID)	*B*. *hermsii* % ID (nearest protein-accession ID)
Uncharacterized protein	AOW96394.1	17	23 (OspC-ABQ42952.1)	50 (Alp-GU784814.1)
Putative lipoprotein	ALN43426.1	35	39 (Putative lipoprotein-AAC67199.1)	62 (Uncharacterized protein-AHH04480.1)
GlpQ	AOW95500.1	39	#	87 (GlpQ-AAT99938.1)
Vsp1	AOW96329.1	22	46 (OspC-AAB86545.1)	62 (Vomp-AHH04378.1)
VlpC2	ALU64348.1	35	37 (VlsE-CAF34025.1)	77 (Vlp19-U52040.1)
VLD2	AOW96264.1	37	37 (VlsE-AAC45764.1)	76 (Vlp42-ABO93439.1)
VlpD1	ALM31565.1	34	37 (VlsE-CAF34025.1)	80 (Vlp-AHH13310.1)
VlpD8	ALU64349.1	36	39 (VlsE-CAH61549.1)	78 (Vlp-ABF82178.1)
VlpD9	ALU64350.1	37	39 (VlsE-CAH61549.1)	84 (Vlp15/16-AAB17735.1)
VlpD10	ALU64352.1	36	36 (VlsE-CAH61549.1)	79 (Vlp-AHH13287.1)

Vlp = variable large protein; Vsp = variable small protein; # = lack of homology compared to *B*. *burgdorferi* proteins; MW = molecular weight; VlsE = Vmp-like sequence expressed; Alp = arthropod associated lipoprotein; Vomp = variable outer membrane protein.

**Figure 5:**
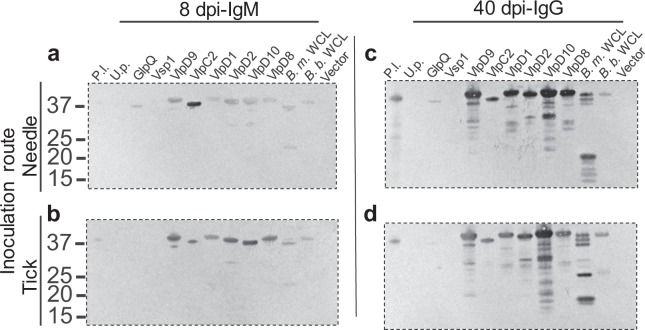
Validation of anti-*B*. *miyamotoi* murine antibodies against recombinant *B*. *miyamotoi* proteins (100 ng). IgM response in CD1 mouse serum collected at 8 dpi to needle (**a**) or tick (**b**) inoculation. IgG response in CD1 mouse serum collected at 40 dpi to needle (**c**) or tick (**d**) inoculation. All sera were diluted 1:200 for immunoblotting. Molecular weight of proteins is expressed in kDa. P.l. = putative lipoprotein; U.p. = uncharacterized protein; Vlp = variable large protein; Vsp = variable small protein; GlpQ = glycerophosphodiester phosphodiesterase; *B*. *m*. lysate = *B*. *miyamotoi* whole cell lysate; *B*. *b*. lysate = *B*. *burgdorferi* whole cell lysate; Vector = empty pETite expression vector. Dashed lines demarcate where the gel has been cropped to exclude molecular weight markers and portions of blots. Uncropped blots blot can be viewed in Supplemental Fig. 2.

The immunoreactivity of selected recombinant proteins was assessed by immunoblotting using mouse anti-*B*. *miyamotoi* sera collected at 8 and 40 dpi (Fig. 5). The IgM response in serum collected from the needle inoculated mouse at 8 dpi resulted in strong reactivity against recombinant VlpC2 (r-VlpC2) and was comparatively weak for r-putative lipoprotein, r-GlpQ, r-VlpD9, r-VlpD1, r-VlpD2, r-VlpD10, and r-VlpD8 (Fig. 5a). In contrast, all six r-Vlps were highly IgM immunoreactive with tick bite inoculated mouse antiserum (Fig. 5b). The r-uncharacterized protein and r-Vsp1 displayed no IgM reactivity with antiserum at 8 dpi regardless of inoculation route (Fig. 5a,b). Reactivity against whole cell lysates (WCL) of *B*. *miyamotoi* (LB-2001) and *B*. *burgdorferi* (B31) was observed at 8 dpi across both needle and tick inoculated sera (Fig. 5a,b).

Antibodies present at 40 dpi (IgG) recognized the entire panel of r-Vlps, independent of infection route. Reactivity against r-GlpQ was present in antiserum from needle inoculated mice, while background antibody recognition was observed in tick inoculation-derived serum (Fig. 5c,d). The r-putative lipoprotein was recognized by antiserum from both needle- and tick-inoculation.  Neither r-Vsp1 nor the r-uncharacterized protein were recognized by serum at the 40 dpi time point (Fig. 5c,d). Additionally, the *B*. *miyamotoi* WCL was highly reactive against antibodies in both groups of serum samples. Recognition was observed, to a lesser extent, in the *B*. *burgdorferi* WCL (Fig. 5c,d).

### Evaluation of cross-reactive anti-*B*. *burgdorferi* serum antibodies against recombinant *B*. *miyamotoi* antigens

**Figure 6:**
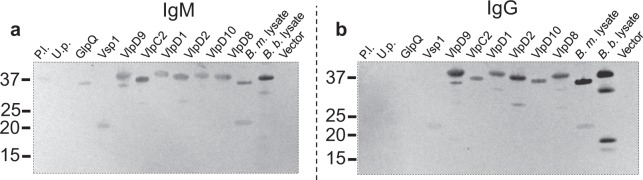
Evaluation of cross-reactive antibodies present in *B*. *burgdorferi*-infected CD1 mice against recombinant *B*. *miyamotoi* proteins (100 ng). Pooled sera collected at 14 dpi from CD1 mice exposed to *B*. *burgdorferi* (B31)-infected *I*. *scapularis* was assayed against *B*. *miyamotoi* recombinant proteins and an IgM (**a**) or IgG (**b**) antibody response observed. Serum was diluted 1:200 for immunoblotting. Molecular weight of proteins is expressed in kDa. P.l. = putative lipoprotein; U.p. = uncharacterized protein; Vlp = variable large protein; Vsp = variable small protein; GlpQ = glycerophosphodiester phosphodiesterase; *B*. *m*. lysate = *B*. *miyamotoi* whole cell lysate*; B*. *b*. lysate = *B*. *burgdorferi* whole cell lysate; Vector = empty pETite expression vector. Dashed lines demarcate where the gel has been cropped to exclude molecular weight markers and portions of blots. Uncropped blots can be viewed in Supplemental Fig. 2.

Anti-*B*. *burgdorferi* sera from mice infected by tick bite (14 dpi) demonstrated strong cross-reactivity (IgM) with regards to all six Vlps *B*. *miyamotoi* recombinants and, to a lesser extent, r-putative lipoprotein, GlpQ, and Vsp1. No cross-reactivity was observed for the r-uncharacterized protein (Fig. 6a). IgG cross-reactivity was observed against all six recombinant Vlps with weak reactivity against Vsp1 (Fig. 6b). Both r-putative lipoprotein and r-GlpQ were negative for IgG cross-reactivity against antibodies present in the *B*. *burgdorferi*-infected murine sera (Fig. 6b). Murine sera antibodies recognized multiple proteins in the *B*. *burgdorferi* WCL and cross-reacted with multiple proteins in the *B*. *miyamotoi* WCL for both IgM and IgG (Fig. 6a,b).

### Reactivity of human BMD and Lyme disease patient serum against recombinant *B*. *miyamotoi* antigens

**Figure 7:**
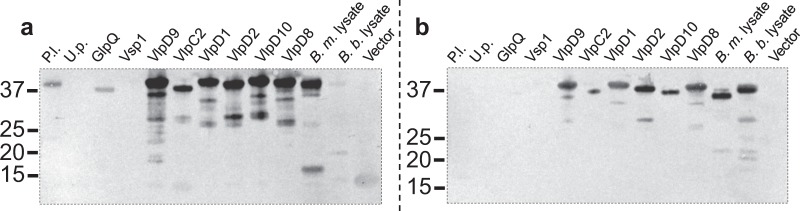
Evaluation of IgG reactivity to *B*. *miyamotoi* recombinant antigens (100 ng) using serum collected from a BMD patient (**a**), and a LD patient (**b**). Patient serum samples diluted 1:200. Molecular weight of proteins is expressed in kDa. P.l. = putative lipoprotein; U.p. = uncharacterized protein; Vlp = variable large protein; Vsp = variable small protein; GlpQ = glycerophosphodiester phosphodiesterase; *B*. *m*. lysate = *B*. *miyamotoi* whole cell lysate; *B*. *burgdorferi* whole cell lysate; Vector = empty pETite expression vector. Dashed lines demarcate where the gel has been cropped to exclude molecular weight markers and portions of blots. Uncropped blots can be viewed in Supplemental Fig. 2.

A BMD patient serum sample blotted against recombinant antigens demonstrated IgG reactivity against all r-Vlps (VlpD9, VlpC2, VlpD1, VlpD2, VlpD10, and VlpD8), the r-putative lipoprotein, and r-GlpQ, but no reactivity against the r-uncharacterized protein or r-Vsp1 (Fig. 7a). Multi-protein reactivity was observed in the *B*. *miyamotoi* WCL but was comparatively weaker in the *B*. *burgdorferi* WCL. IgG serum antibodies from a LD patient demonstrated cross-reactivity against all r-Vlps but was absent for r-uncharacterized protein, r-GlpQ, and r-Vsp1. Significantly, there was also no cross-reactivity against the r-putative lipoprotein. Both *B*. *burgdorferi* and *B*. *miyamotoi* WCLs demonstrated reactive proteins (Fig. 7b).

## Discussion

Accurate diagnosis of BMD continues to warrant greater attention as the impact on human disease burden increases. Since its emergence as a human pathogen in 2011, cases have been described across multiple continents where ticks of the *Ixodes* genera are present, however, approaches to generate diagnostics to distinguish *B*. *miyamotoi* infections from other borrelioses are only beginning to be investigated^5,7,13,19–22^. The present work took an immunoproteomic approach to compare host acute and convalescent antibody responses against *B*. *miyamotoi* infection by either needle or tick vector inoculation for the identification of novel serodiagnostic targets.

Infection in murine hosts produced differential antigen recognition patterns dependent upon infection duration (8 and 40 dpi) and inoculation route (needle vs. tick bite). IgM antibodies (8 dpi) were reactive against a number of proteins previously identified during *B*. *miyamotoi* infection including: GlpQ, Vsp1, and 9 Vlps (VlpC2, D2, D1, D8, D9, D10, 5, 15/16, and one undesignated Vlp)^11,14,23^. Antibody responses to multiple Vlps suggests common epitope recognition, tandem expression, or borrelial serotype switching during acute infection. Additionally, this could also be indicative of the clonality, or lack thereof, concerning strains used for infection. *Borrelia miyamotoi*-infected *I*. *scapularis* used in this study originated from field collection^24^. Consequently, murine infection may represent a non-clonal strain of *B*. *miyamotoi*, contributing to the expression of multiple Vlps, which may have important implications on observed antibody response.

GlpQ, Vsp1, and multiple Vlps were again recognized at 40 dpi, suggesting their utility as serodiagnostic targets with both IgM and IgG antibody responses. A greater number of antigens were recognized by the IgG response at 40 dpi relative to the IgM response at 8 dpi indicating the maturity of the humoral response with time. Redundancy in protein identification from these spots was prevalent; however, unique identifications included proteins grouped with cellular components, molecular functions, biological processes, and several hypothetical proteins with unclassified function (listed in Table 1).

Immunoreactive spots were more abundant in 2DE immunoblots probed with antisera from tick bite inoculated mice compared to needle inoculated mice indicative of differential protein expression profile. Alternatively, immunoreactivity against needle inoculated *B*. *miyamotoi* LB-2001 could reflect multiple passages through SCID mice prior to stable *in vitro* cultivation^25–27^. Although there are no direct data regarding *B*. *miyamotoi* plasmid loss or gene rearrangement upon serial passage, it has been posited as an underlying reason for gene loss in at least one study^28^. Plasmid content representative of the strains utilized in this study was not compared but may also play a role in the observed results. However, it has been firmly established that environmental factors such as temperature and pH, which differ in the tick versus *in vitro* cultivation, impact *B*. *burgdorferi* gene expression profiles providing rationale for investigations into gene regulation during tick transmission^29–33^. For example, the spots present in the immunoblots probed with antisera from tick bite inoculated mice but absent in needle inoculated mice may offer clues to determine borrelial gene products essential for survival, maintenance, and dissemination in ticks.

Assessment of various Vmps in conjunction with GlpQ to improve serodiagnosis of BMD suggests that multi-antigenic detection yields higher sensitivity and specificity^14^. Our data supported those findings by demonstrating recognition of r-Vlps (VlpD9, C2, D2, D10, and D8) with mouse anti-*B*. *miyamotoi* antibodies and with human BMD antiserum. However, the Vlps were similarly recognized with antisera from *B*. *burgdorferi*-infected mice and the human LD patient suggesting that serological utility of these antigens may be compromised as a reliable diagnostic tool. The observed cross-reactivity is possibly due to orthology to *B*. *burgdorferi* proteins VlsE and OspC, and is especially notable as the 6^th^ invariant region (IVR6) of VlsE comprises the C6 antigen that is used extensively in diagnostic testing for LD^16,17^. It is noteworthy that both the BMD and LD patient serum used in this study were C6-positive. Indeed, recent reports have indicated that BMD patient serum samples react positively in C6 antigen testing^12,34^. Thus, previous or active infections with BMD or LD cannot be excluded for patient sera used in this or future studies where observable reactivity against Vlps or alternative antigens is noted.

Recombinant Vsp1 and the uncharacterized protein were not reactive against the mouse antisera used in the 2DE immunoblot discovery and, therefore, were not confirmed as immunogens in our mouse model. This result may be due to the presence of multiple overlapping non-immunogenic proteins identified within a single immunoreactive spot. The uncharacterized protein was most closely homologous to the *B*. *hermsii* Alp protein^18^. Like the *B*. *miyamotoi* uncharacterized protein, Alp did not elicit an antibody response in infected mice and was postulated to be associated with the tick environment. Curiously, the lack of recognition of our recombinant Vsp1 with mouse antiserum conflicted with the findings of Wagemakers *et al*. who described Vsp1 as an immunodominant antigen from their *B*. *miyamotoi* strain LB-2001 needle inoculated mice^11^. However, those investigators injected their mice with a dose of organisms 3 logs higher than that used in our study (10^7^ vs 10^4^) perhaps accounting for the observed differences. However, these researchers did observe Vmp switching from dominant expression of Vsp1 to VlpC2 in acute infection^11^. Our infection model resulted in dominant, acute expression of VlpC2, possibly due to Vmp switching brought about by differences in cultivation conditions or infection kinetics between studies. Similarly, we did not observe an anti-Vsp1 response from tick bite inoculated mice whereas the Wagemakers *et al*. study found that only 2 of 9 human BMD (presumably from tick bite infection) patient serum samples were positive for Vsp1^11^. Our preliminary results demonstrated no Vsp1 reactivity in BMD or LD human sera. Though recent studies have shown its utility as a specific diagnostic marker, there could exist disparity among *B*. *miyamotoi* strains originating in North American versus Europe^13^. These results illustrate the complexity of differential Vmp gene expression and the corresponding role of host seroconversion dependent on inoculation route which awaits further study. Interestingly, reactivity against r-GlpQ was seen by IgG in serum from needle inoculated mice at 40 dpi, and was weakly recognized in serum from mice infected by tick inoculation. This result suggests differential upregulation between inoculation routes and/or differences in antibody responses between reservoir vs. incidental (human) hosts that warrants further investigation.

The putative lipoprotein reacted strongly against the anti-*B*. *miyamotoi* antibodies raised in both the needle and tick inoculated mice, emerging as a novel candidate serodiagnostic antigen. The putative lipoprotein has a calculated molecular mass of 35 kDa and is encoded by a gene localized to the lpD plasmid of *B*. *miyamotoi* strain LB-2001^26^. The homologous protein in *B*. *miyamotoi* CT13-2396 has 80% amino acid identity^35^ and orthologs are reported in GenBank for various RFB (including *B*. *hermsii*, *B*. *turicatae*, *B*. *crociduriae*, and *B*. *duttonii*) with amino acid identities of approximately 55% with *B*. *miyamotoi*. Amino acid alignment revealed regions of identity (approximately 39%) with a *B*. *burgdorferi* putative lipoprotein. The function or role in pathogenesis of this *B*. *miyamotoi* putative lipoprotein is unknown. Preliminary data regarding the r-putative lipoprotein displayed background cross-reactivity in *B*. *burgdorferi*-infected mice via IgM, but not IgG. Lack of IgG response was also noted in our singular LD human serum. Importantly, antibody response directed against the putative lipoprotein was dominant in the BMD convalescent human serum sample, thereby, providing preliminary evidence for serological distinction between BMD and LD. Antigenic cross-reactivity with the putative lipoprotein against antibodies directed to other RFB remains to be determined. A limitation of current work includes the number of human serum samples, especially those which are of similar infection status across disease groups (i.e. LD vs. BMD) and will be the subject of future studies. Additional human serum samples from BMD, LD, and soft tick-borne RFB patients will be necessary in the appraisal of this putative lipoprotein as a serodiagnostic target.

In conclusion, we used an immunoproteomic approach to identify *B*. *miyamotoi* antigens that induce the host antibody response post-infection. Our results indicated that although Vmps are immunodominant, agreeing with other reports, antigenic cross-reactivity with anti-*B*. *burgdorferi* antibodies may be problematic. The putative lipoprotein has emerged as a potential serodiagnostic candidate to augment BMD testing with GlpQ and Vmps. Multiple proteins revealed as immunogenic in this study may aid in the development of new serodiagnostic tests for BMD.

## Methods and Materials

### *Borrelia miyamotoi* culture and mouse infections

Low passage (≤4) *B*. *miyamotoi* strain LB-2001^36^, (kindly supplied by Joppe Hovius, Center for Experimental and Molecular Medicine, Amsterdam, The Netherlands) was cultivated at 34 °C with 5% CO_2_ in modified Kelly-Pettenkofer medium (MKP-F) as previously described^27,37^. Spirochetes were visualized using dark field microscopy and enumerated with a Cellometer (Nexcelom, Lawrence, MA). Outbred, female CD-1 mice (n = 5) (Charles River, Wilmington, MA) 6–8 weeks of age were needle inoculated subcutaneously with a 100 µl suspension of *B*. *miyamotoi* (1 × 10^4^) in MKP-F. A separate cohort of CD-1 mice (n = 3–5) were infested with *B*. *miyamotoi*-infected *I*. *scapularis* immatures (i.e. larvae or nymphs) that originated from naturally infected female ticks collected in Minnesota that passed infection to their offspring. The tick feeding protocol was previously described^38^ followed by serum collection at 8 or 40 dpi. Serum from two CD-1 mice infested with nymphal *B*. *burgdorferi* (strain B31)-infected *I*. *scapularis* was collected 14 dpi and pooled. All murine infections were performed once. Animal experiments were approved and conducted in accordance with guidelines and regulations as established by the Division of Vector-Borne Diseases Institutional Animal Care and Use Committee (IACUC) protocol number 16–020.

### Preparation of borrelial lysate for 2DE

Mid to late log-phase *Borrelia* were collected via centrifugation at 4000 × g for 15 min at 4 °C with approximately 2.6 × 10^8^ spirochetes/ml used as starting material for protein preparation. Bacterial pellets were washed twice in sterile 1X PBS and stored at −80 °C prior to use. Pellets were resuspended in 10 ml of 20 mM Tris☐HCl (pH 8.0) containing 30 µg DNaseI (Thermo Scientific, Rockford, IL), 30 µg RNaseA, and 1 Complete Protease Inhibitor (PIC) tablet (Roche Applied Sciences, Indianapolis, IN). Bacterial suspension was sonicated on ice using a Branson Sonifier 450 (Branson Ultrasonics Corporation, Danbury, CT) for 60 s followed by a 60 s rest a total of ten times using the following settings: 50% duty, output-5. The cell lysate was separated from unlysed cell debris by centrifugation at 4500 × g for 20 min at 4 °C. Membrane-associated proteins were pelleted using a Sorvall WX Ultra Series ultracentrifuge (Thermo Electron Corporation) at 100,000 × g for 1 h at 4 °C resuspended in 20 mM Tris (pH 8.0) containing DNaseI, RNaseA, and PIC (as specified above), and incubated at 4 °C overnight (O/N). The protein suspension was dialyzed against 10 mM ammonium bicarbonate O/N at 4 °C in a Slide-A-Lyzer G2 cassette (Thermo Scientific, Rockford, IL) and protein concentration determined by BCA (Thermo Scientific, Rockford, IL). Protein aliquots (100 µg) were lyophilized using a Labconco Freezone 4.5 (Labconco, Kansas City, MO) and stored at −80 °C.

### 2DE polyacrylamide gel electrophoresis

Membrane-associated protein samples (100 µg) were solubilized in 8 M urea, 2 M thiourea, 60 mM DTT, 4% CHAPS, and 1% ASB-14. Ampholytes were added to a final concentration of 0.7% pH 4–7 and 0.3% pH 3–10 for pH 4–7 strips or 0.5% pH 6–9 and 0.5% pH 9–11 for pH 6–11 strips. Samples were solubilized for 72 hours at 4 °C followed by O/N application at room temperature (RT) to 7 cm pH 4–7 or pH 6–11 Immobiline dry strips (GE Healthcare, Piscataway, NJ). Isoelectric focusing (IEF) was performed on a GE Multiphor II (GE Healthcare, Piscataway, NJ) using the following settings: 50, 100, 150, 200, 250, and 300 V for 6 minutes, 500 V for 12 minutes, and 3000 V for 5 hours. Focused strips were equilibrated for 15 minutes at RT in two sequential washes of 6 M urea, 2% SDS, 0.05 M Tris·HCl (pH), and 50% glycerol containing either 2% DTT or 2.5% iodoacetamide for respective incubations. Proteins were resolved in the second dimension using 4–12% Bis-Tris SDS-polyacrylamide gels (Life Technologies, Carlsbad, CA). Gels were silver stained for protein identification (Pierce Silver Stain for Mass Spectrometry, Thermo Scientific, Rockland, IL) or transferred to nitrocellulose or PVDF for immunoblotting.

### Trypsin digestion and mass spectrometry

Proteins within destained gel slices were dried in a SpeedVac (Savant, Thermo Scientific, Rockland, IL) followed by resuspension in 83 ng/µl of trypsin (Promega, Madison, WI) in 0.2 M ammonium bicarbonate and incubated O/N at 37 °C. Reaction was terminated with the addition of 10% (v/v) trifluoroacetic acid (TFA). Peptides were extracted by suspending gel slices in 0.1% TFA/60% acetonitrile (ACN) (v/v), incubated at 37 °C for 40 minutes, dried via SpeedVac and identified by LC-MS/MS at the Proteomics and Metabolomics Facility, Colorado State University, Fort Collins, Colorado. Peptides (0.5 µg) were resuspended in 5% ACN/0.1% formic acid and applied to an on-line enrichment column (Waters Symmetry Trap C18 100 Å, 5 µm, 180 µm ID × 20 mm column) with chromatographic separation via reverse phase nanospray column (Waters, peptide BEH C18; 17 µm, 75 µm ID × 100 mm column, 45 °C) with a 30 minute gradient: 3–8% buffer B (0.1% formic acid in ACN) over 3 minutes followed by 8–35% buffer B over 27 minutes at a flow rate of 350 nanoliters/minute. Peptides were injected into an Orbitrap Velos mass spectrometer (Thermo Scientific, Rockford, IL) equipped with a Nanospray Flex ion source (Thermo Scientific, Rockford, IL). A *m/z* range of 400–2000 of spectra were gathered in positive mode ionization with a dynamic exclusion limit of 2 MS/MS spectra of a given *m/z* value for 30 s (exclusion duration of 90 s). For pH 6–11, ions with a charge state of +2 or +3 were accepted for MS/MS. The instrument was operated in FT mode for MS detection (resolution of 60,000) and ion trap mode for MS/MS detection with a normalized collision energy set to 35%. Resulting tandem mass spectra were extracted by ProteoWizard MsConvert (version 3.0). Spectra were searched in Mascot (Matrix Science) with a UniProt database for *B*. *miyamotoi*. No amino acid modifications were searched for proteins identified in the pH range of 4–7. For pH 6–11, oxidation of methionine and carbamidomethyl of cysteine amino acid modifications were viewed. Peptide and protein identities were viewed in Scaffold (version 4.8.7). Identities were accepted with a >90% peptide and >99% protein probability threshold and at least two unique peptides per protein identity (Supplemental Table 1). Mass spectrometry was performed for all spots at least once, with some identified twice.

### Production of recombinant proteins and immunoblotting

Genes were amplified by PCR from *B*. *miyamotoi* genomic DNA (gDNA) using primers listed in Table 3, cloned into pETite N-His vector (Lucigen, Middleton, WI), and transformed into *E*. *coli* BL21 (DE3) according to the manufacturer’s instructions. Recombinant proteins were expressed and purified using a QiaExpress Ni-NTA FastStart kit (Qiagen, Valencia, CA) as described previously^39^. Proteins (100 ng/individual protein) were transferred to PVDF membranes for immunoblotting with mouse or human serum samples (1:200), followed by incubation with alkaline phosphatase conjugated goat anti-mouse or goat anti-human IgM or IgG (1:5000) with development by NBT/BCIP with similar approximated reaction times across all blots according to standard procedures. All recombinant protein immunoblots were performed twice.

**Table 3:** Primers used for gene amplification for pETite expression vector cloning.

Expression product	Forward primer	Reverse primer
Uncharacterized protein	CATCATCACCACCATCACAAAGATAATGCTACTGAATAC	GTGGCGGCCGCTCTATTATGGTGCTGATAGTATGTCTCG
Putative lipoprotein	CATCATCACCACCATCACAGTAATTTGATATCTCAGGAG	GTGGCGGCCGCTCTATTATCTTTCAAGAGTCCTCTAAAC
GlpQ	CATCATCACCACCATCACTTTAAACAAGAAATGGGTGAAAAC	GTGGCGGCCGCTCTATTATATGAAATTCATTACTGTGTCAGT
Vsp1	CATCATCACCACCATCACCCGGCACCTAAGGAAGGGCAG	GTGGCGGCCGCTCTATTATGAAGATTGACCAGCAGTTAA
VlpC2	CATCATCACCACCATCACACTTTATTTTTAGGATTAGTG	GTGGCGGCCGCTCTATTATTCACTTTTAGTTTCAGGTGT
VlpD2	CATCATCACCACCATCACGGAGGGGAAGATCCACAAAAG	GTGGCGGCCGCTCTATTAGTTTTGTGCACTAGCTGTTACTTC
VlpD1	CATCATCACCACCATCACACTTTATTTTTAATAATAGGATGTAATAAT	GTGGCGGCCGCTCTATTAGTTTTGTACACTAGTTGTTGATTC
VlpD8	CATCATCACCACCATCACGGAGGGGAAGATCCACAA AAG	GTGGCGGCCGCTCTATTAGTTTTGTACACTAGTTGTTGCTTCTGC
VlpD9	CATCATCACCACCATCACGGAGGGGAAGATCCACAAAAG	GTGGCGGCCGCTCTATTAGTTTTGTACACTAGCTGTTACTTC
VlpD10	CATCATCACCACCATCACGGAGGGGAAGATCCACAAAAG	GTGGCGGCCGCTCTATTAGTTTTGTGCACTAGCTGTTAC

Vsp = variable small protein; Vlp = variable large protein; underlined nucleotides denote sequences used for expression vector insertion.

### Patient samples

Two human serum samples, obtained from New York Medical College, were from one patient with BMD and a second with LD. A case summary for the BMD patient has been published^34^. The BMD patient presented to the clinic approximately one month post-symptom onset with a history of tick exposure and two documented episodes of fever relapse. The blood sample tested from that clinic was positive by PCR for *B*. *miyamotoi*; the serum utilized in the current study was collected approximately 100 days after onset of the patient’s symptoms (i.e. 70 days after confirmation of the diagnosis by PCR). Serologic testing for LD performed at New York Medical College was positive by the whole cell sonicate (WCS) Captia^TM^ *Borrelia burgdorferi* IgG/IgM enzyme immune assay (EIA; Trinity Biotech, Jamestown, NY) and by the C6 Lyme ELISA (Oxford Immunotec, Marlborough, MA), but negative by MarDx^®^ *B*. *burgdorferi* IgM and IgG Marblot western blots (Trinity Biotech).

The LD patient reported tick exposure and presented to the clinic with a single erythema migrans skin lesion with duration of one day. A skin biopsy was culture and PCR-positive for *B*. *burgdorferi*. Serologic testing was performed at the CDC using the WCS EIA (Vidas Lyme IgM and IgG Polyvalent Assay, bio-Mérieux, Inc., Durham, NC), the C6 Lyme ELISA (Oxford Immunotec, Marlborough, MA) and the MarDx^®^ *B*. *burgdorferi* IgM and IgG Marblot western blots (Trinity Biotech). The patient was positive for LD by standard two-tiered testing (C6 ELISA/IgM western blot) and by 2-EIA criteria (WCS EIA/C6 ELISA)^40^. Serum was collected 22 days after the patient initiated doxycycline therapy. All methods regarding human samples were approved and carried out in accordance with the guidelines and regulations set forth by the Institutional Review Board of the Centers for Disease Control and Prevention and New York Medical College. Both patients provided informed consent.

## Supplementary information

Supplementary material
