# Supplementary material for: Immunoproteomic analysis of Borrelia miyamotoi for the identification of serodiagnostic antigens
Source: Sci Rep. 2019 Nov 14;9:16808. doi: 10.1038/s41598-019-53248-5 (PMC6856195; doi:10.1038/s41598-019-53248-5)
Supplement: Supplementary file 1 — Supplementary material [file 41598_2019_53248_MOESM1_ESM.docx]

**Supplementary material**

**Immunoproteomic analysis of *Borrelia miyamotoi* for the identification of serodiagnostic antigens**

Emma K. Harris, Marisa R. Harton, Maria Angela de Mello Marques, John T. Belisle, Claudia R. Molins, Nicole Breuner, Gary P. Wormser, Robert D. Gilmore#

#Address correspondence to Robert D. Gilmore rbg9@cdc.gov

**Table 1.** Raw LC-MS/MS Scaffold protein identities corresponding to immunoreactive spots. Each spot number (no.) corresponds to respective unique and full peptide and spectrum detected during LC-MS/MS.

| **Spot no.** | **Protein name** | **NCBI Accession ID** | **UniProt Accession ID** | **Protein molecular weight (Da)** | **Protein identification probability** | **Exclusive unique peptide count** | **Exclusive unique spectrum count** | **Exclusive spectrum count** | **Percentage of total spectra** | **Percentage sequence coverage** | |
| --- | --- | --- | --- | --- | --- | --- | --- | --- | --- | --- | --- |
| 1 | Flagellin OS=*Borrelia miyamotoi* GN=AXH25_00725 PE=3 SV=1 | AGT27144.1 | A0A1D8TCQ1\|A0A1D8TCQ1_9SPIR | 35445.9 | 100.00% | 11 | 15 | 196 | 2.14% | 41.90% | |
| 1 | Glycerophosphodiester phosphodiesterase OS=*Borrelia miyamotoi* GN=AXH25_01180 PE=4 SV=1 | AGT27237.1 | A0A1D8TD05\|A0A1D8TD05_9SPIR | 38703.2 | 100.00% | 16 | 20 | 203 | 0.45% | 13.40% | |
| 1 | Phosphate ABC transporter substrate-binding protein OS=*Borrelia miyamotoi* GN=AXH25_01045 PE=4 SV=1 | AGT27210.1 | A0A1D8TDH6\|A0A1D8TDH6_9SPIR | 31380.9 | 100.00% | 2 | 4 | 41 | 2.21% | 60.20% | |
| 1 | Variable large protein OS=*Borrelia miyamotoi* OX=47466 GN=vlpC2 PE=4 SV=1 | ALU64348.1 | A0A109PHQ5_9SPIR,  A0A1D8TFE5_9SPIR | 34513.6 | 100.00% | 26 | 32 | 5511 | 33.90% | 73.30% | |
| 2 | 60 kDa chaperonin OS=*Borrelia miyamotoi* GN=groL PE=3 SV=1 | AGT27591.1 | A0A1D8TE31\|A0A1D8TE31_9SPIR | 58966.8 | 100.00% | 5 | 5 | 11 | 0.10% | 12.80% | |
| 2 | Flagellar protein OS=*Borrelia miyamotoi* GN=AXH25_03295 PE=4 SV=1 | AGT27611.1 | A0A1D8TE06\|A0A1D8TE06_9SPIR | 39285.2 | 100.00% | 10 | 10 | 95 | 0.28% | 21.10% | |
| 2 | Flagellin OS=*Borrelia miyamotoi* GN=AXH25_00725 PE=3 SV=1 | AGT27144.1 | A0A1D8TCQ1\|A0A1D8TCQ1_9SPIR | 35445.9 | 100.00% | 13 | 29 | 855 | 8.89% | 45.80% | |
| 2 | Glycerophosphodiester phosphodiesterase OS=*Borrelia miyamotoi* GN=AXH25_01180 PE=4 SV=1 | AGT27237.1 | A0A1D8TD05\|A0A1D8TD05_9SPIR | 38703.2 | 100.00% | 10 | 11 | 39 | 0.05% | 14.60% | |
| 2 | Ornithine carbamoyltransferase OS=*Borrelia miyamotoi* GN=AXH25_04195 PE=3 SV=1 | WP_043867909.1 | A0A1D8TEM8\|A0A1D8TEM8_9SPIR | 36921.7 | 100.00% | 7 | 7 | 27 | 0.11% | 10.60% | |
| 2 | Phosphoglycerate kinase OS=*Borrelia miyamotoi* GN=pgk PE=3 SV=1 | AGT27059.1 | A0A1D8TCM0\|A0A1D8TCM0_9SPIR | 42574.7 | 100.00% | 4 | 4 | 10 | 0.41% | 34.40% | |
| 2 | Variable large protein OS=*Borrelia miyamotoi* GN=AXH25_04660 PE=4 SV=1 | ALU64349.1 | A0A1D8TFY1\|A0A1D8TFY1_9SPIR | 35732.8 | 100.00% | 2 | 2 | 2 | 6.10% | 45.80% | |
| 2 | Variable large protein OS=*Borrelia miyamotoi* GN=AXH25_04805 PE=4 SV=1 | AOW96300.1 | A0A1D8TFA1\|A0A1D8TFA1_9SPIR | 34611.9 | 100.00% | 2 | 2 | 9 | 0.02% | 4.90% | |
| 2 | Variable large protein OS=*Borrelia miyamotoi* GN=AXH25_04900 PE=4 SV=1 | ALM31567.1 | A0A1D8TF85\|A0A1D8TF85_9SPIR | 36693 | 100.00% | 3 | 3 | 6 | 0.11% | 11.20% | |
| 3 | ABC transporter substrate-binding protein OS=*Borrelia miyamotoi* OX=47466 GN=AXH25_01615 PE=4 SV=1 | AGT27324.1 | A0A1D8TD60_9SPIR | 61010.9 | 100.00% | 32 | 56 | 2597 | 0.08% | 5.50% | |
| 3 | ABC transporter substrate-binding protein OS=*Borrelia miyamotoi* OX=47466 GN=AXH25_01610 PE=4 SV=1 | AGT27323.1 | A0A1D8TD91_9SPIR | 60830.7 | 100.00% | 18 | 25 | 513 | 1.15% | 32.90% | |
| 3 | Variable large protein OS=*Borrelia miyamotoi* OX=47466 GN=vlpC2 PE=4 SV=1 | ALU64348.1 | A0A109PHQ5_9SPIR,  A0A1D8TFE5_9SPIR | 34513.6 | 100.00% | 5 | 5 | 39 | 0.07% | 18.80% | |
| 4 | 60 kDa chaperonin OS=*Borrelia miyamotoi* GN=groL PE=3 SV=1 | AGT27591.1 | A0A1D8TE31\|A0A1D8TE31_9SPIR | 58966.8 | 100.00% | 10 | 13 | 68 | 0.07% | 8.68% | |
| 4 | DNA-directed RNA polymerase subunit alpha OS=*Borrelia miyamotoi* GN=rpoA PE=3 SV=1 | AGT27462.1 | A0A1D8TDR4\|A0A1D8TDR4_9SPIR | 38142.3 | 100.00% | 17 | 26 | 312 | 19.30% | 66.30% | |
| 4 | Enolase OS=*Borrelia miyamotoi* GN=eno PE=3 SV=1 | AGT27330.1 | A0A1D8TDA6\|A0A1D8TDA6_9SPIR | 47094.7 | 100.00% | 3 | 3 | 9 | 3.82% | 41.40% | |
| 4 | Flagellin OS=*Borrelia miyamotoi* GN=AXH25_00725 PE=3 SV=1 | AGT27144.1 | A0A1D8TCQ1\|A0A1D8TCQ1_9SPIR | 35445.9 | 100.00% | 9 | 16 | 102 | 5.48% | 35.90% | |
| 4 | Putative lipoprotein OS=*Borrelia miyamotoi* GN=AXH25_04705 PE=4 SV=1 | ALN43426.1 | A0A0U2RX32\|A0A0U2RX32_9SPIR | 35313.8 | 100.00% | 2 | 2 | 11 | 0.04% | 10.50% | |
| 4 | Uncharacterized protein OS=*Borrelia miyamotoi* GN=AXH25_03690 PE=4 SV=1 | AGT27688.1 | A0A1D8TE76\|A0A1D8TE76_9SPIR | 77129.2 | 100.00% | 4 | 5 | 22 | 0.41% | 18.30% | |
| 4 | Variable large protein OS=*Borrelia miyamotoi* GN=AXH25_04660 PE=4 SV=1 | ALU64349.1 | A0A1D8TFY1\|A0A1D8TFY1_9SPIR | 35732.8 | 100.00% | 29 | 41 | 746 | 0.70% | 25.30% | |
| 4 | Variable large protein OS=*Borrelia miyamotoi* GN=AXH25_04665 PE=4 SV=1 | ALU64350.1 | A0A1D8TFG6\|A0A1D8TFG6_9SPIR | 36578.6 | 100.00% | 2 | 2 | 7 | 3.22% | 59.50% | |
| 4 | Variable large protein OS=*Borrelia miyamotoi* GN=AXH25_04680 PE=4 SV=1 | AOW96282.1 | A0A1D8TF92\|A0A1D8TF92_9SPIR | 35795.1 | 100.00% | 5 | 5 | 22 | 0.09% | 9.98% | |
| 4 | Variable large protein OS=*Borrelia miyamotoi* GN=AXH25_04900 PE=4 SV=1 | ALM31567.1 | A0A1D8TF85\|A0A1D8TF85_9SPIR | 36693 | 100.00% | 5 | 5 | 21 | 1.05% | 36.20% | |
| 4 | Variable large protein OS=*Borrelia miyamotoi* GN=AXH25_06025 PE=4 SV=1 | ALM31565.1 | A0A1D8TF59\|A0A1D8TF59_9SPIR | 34299 | 100.00% | 4 | 4 | 18 | 0.11% | 8.68% | |
| 5 | 60 kDa chaperonin OS=*Borrelia miyamotoi* GN=groL PE=3 SV=1 | AGT27591.1 | A0A1D8TE31\|A0A1D8TE31_9SPIR | 58966.8 | 100.00% | 4 | 4 | 14 | 0.23% | 12.60% | |
| 5 | DNA-directed RNA polymerase subunit alpha OS=*Borrelia miyamotoi* GN=rpoA PE=3 SV=1 | AGT27462.1 | A0A1D8TDR4\|A0A1D8TDR4_9SPIR | 38142.3 | 100.00% | 16 | 25 | 350 | 7.69% | 72.00% | |
| 5 | Enolase OS=*Borrelia miyamotoi* GN=eno PE=3 SV=1 | AGT27330.1 | A0A1D8TDA6\|A0A1D8TDA6_9SPIR | 47094.7 | 100.00% | 2 | 2 | 3 | 0.07% | 22.20% | |
| 5 | Flagellin OS=*Borrelia miyamotoi* GN=AXH25_00725 PE=3 SV=1 | AGT27144.1 | A0A1D8TCQ1\|A0A1D8TCQ1_9SPIR | 35445.9 | 100.00% | 10 | 15 | 131 | 0.23% | 35.10% | |
| 5 | Glycerophosphodiester phosphodiesterase OS=*Borrelia miyamotoi* GN=AXH25_01180 PE=4 SV=1 | AGT27237.1 | A0A1D8TD05\|A0A1D8TD05_9SPIR | 38703.2 | 100.00% | 2 | 2 | 4 | 0.22% | 30.30% | |
| 5 | Putative lipoprotein OS=*Borrelia miyamotoi* GN=AXH25_04705 PE=4 SV=1 | ALN43426.1 | A0A0U2RX32\|A0A0U2RX32_9SPIR | 35313.8 | 100.00% | 3 | 3 | 16 | 0.19% | 24.90% | |
| 5 | Variable large protein OS=*Borrelia miyamotoi* GN=AXH25_04660 PE=4 SV=1 | ALU64349.1 | A0A1D8TFY1\|A0A1D8TFY1_9SPIR | 35732.8 | 100.00% | 32 | 48 | 1613 | 0.15% | 13.90% | |
| 5 | Variable large protein OS=*Borrelia miyamotoi* GN=AXH25_04665 PE=4 SV=1 | ALU64350.1 | A0A1D8TFG6\|A0A1D8TFG6_9SPIR | 36578.6 | 100.00% | 3 | 4 | 31 | 3.63% | 52.50% | |
| 5 | Variable large protein OS=*Borrelia miyamotoi* GN=AXH25_04680 PE=4 SV=1 | AOW96282.1 | A0A1D8TF92\|A0A1D8TF92_9SPIR | 35795.1 | 100.00% | 7 | 10 | 129 | 0.03% | 6.03% | |
| 5 | Variable large protein OS=*Borrelia miyamotoi* GN=AXH25_04900 PE=4 SV=1 | ALM31567.1 | A0A1D8TF85\|A0A1D8TF85_9SPIR | 36693 | 100.00% | 9 | 9 | 56 | 1.36% | 41.30% | |
| 5 | Variable large protein OS=*Borrelia miyamotoi* GN=AXH25_06025 PE=4 SV=1 | ALM31565.1 | A0A1D8TF59\|A0A1D8TF59_9SPIR | 34299 | 100.00% | 8 | 10 | 90 | 0.04% | 7.49% | |
| 5 | Variable large protein OS=*Borrelia miyamotoi* GN=vlpD10 PE=4 SV=1 | ALU64352.1 | A0A0X8ASK8\|A0A0X8ASK8_9SPIR | 36184 | 100.00% | 2 | 3 | 6 | 0.17% | 11.30% | |
| 6 | DNA-directed RNA polymerase subunit alpha OS=Borrelia miyamotoi GN=rpoA PE=3 SV=1 | AGT27462.1 | A0A1D8TDR4\|A0A1D8TDR4_9SPIR | 38142.3 | 100.00% | 15 | 21 | 181 | 16.70% | 70.30% | |
| 6 | Flagellar assembly protein FliH OS=*Borrelia miyamotoi* GN=AXH25_01425 PE=4 SV=1 | AGT27286.1 | A0A1D8TD19\|A0A1D8TD19_9SPIR | 35109.5 | 100.00% | 14 | 22 | 189 | 0.32% | 16.80% | |
| 6 | Flagellin OS=Borrelia miyamotoi GN=AXH25_00725 PE=3 SV=1 | AGT27144.1 | A0A1D8TCQ1\|A0A1D8TCQ1_9SPIR | 35445.9 | 100.00% | 12 | 28 | 735 | 1.34% | 42.30% | |
| 6 | Glycerophosphodiester phosphodiesterase OS=*Borrelia miyamotoi* GN=AXH25_01180 PE=4 SV=1 | AGT27237.1 | A0A1D8TD05\|A0A1D8TD05_9SPIR | 38703.2 | 100.00% | 13 | 15 | 112 | 0.58% | 46.50% | |
| 6 | Putative lipoprotein OS=Borrelia miyamotoi GN=AXH25_04705 PE=4 SV=1 | ALN43426.1 | A0A0U2RX32\|A0A0U2RX32_9SPIR | 35313.8 | 100.00% | 4 | 4 | 24 | 0.93% | 39.60% | |
| 6 | Uncharacterized protein OS=*Borrelia miyamotoi* GN=AXH25_04255 PE=4 SV=1 | AOW96351.1 | A0A1D8TFD7\|A0A1D8TFD7_9SPIR | 34906 | 100.00% | 6 | 7 | 32 | 0.06% | 13.10% | |
| 6 | Variable large protein OS=*Borrelia miyamotoi* GN=AXH25_04660 PE=4 SV=1 | ALU64349.1 | A0A1D8TFY1\|A0A1D8TFY1_9SPIR | 35732.8 | 100.00% | 22 | 30 | 342 | 1.80% | 54.20% | |
| 6 | Variable large protein OS=*Borrelia miyamotoi* GN=AXH25_04665 PE=4 SV=1 | ALU64350.1 | A0A1D8TFG6\|A0A1D8TFG6_9SPIR | 36578.6 | 100.00% | 5 | 6 | 59 | 0.07% | 6.09% | |
| 6 | Variable large protein OS=*Borrelia miyamotoi* GN=AXH25_04680 PE=4 SV=1 | AOW96282.1 | A0A1D8TF92\|A0A1D8TF92_9SPIR | 35795.1 | 100.00% | 7 | 11 | 204 | 1.88% | 52.90% | |
| 6 | Variable large protein OS=*Borrelia miyamotoi* GN=AXH25_04900 PE=4 SV=1 | ALM31567.1 | A0A1D8TF85\|A0A1D8TF85_9SPIR | 36693 | 100.00% | 14 | 23 | 393 | 7.30% | 45.80% | |
| 6 | Variable large protein OS=Borrelia miyamotoi GN=AXH25_06025 PE=4 SV=1 | ALM31565.1 | A0A1D8TF59\|A0A1D8TF59_9SPIR | 34299 | 100.00% | 11 | 14 | 284 | 0.71% | 32.90% | |
| 6 | Variable large protein OS=*Borrelia miyamotoi* GN=vlpD10 PE=4 SV=1 | ALU64352.1 | A0A0X8ASK8\|A0A0X8ASK8_9SPIR | 36184 | 100.00% | 3 | 7 | 74 | 1.11% | 53.30% | |
| 6 | Variable small protein 1 OS=*Borrelia miyamotoi* GN=AXH25_04685 PE=4 SV=1 | AJA67245.2 | A0A1D8TFC5\|A0A1D8TFC5_9SPIR | 21956.2 | 100.00% | 3 | 4 | 43 | 0.24% | 18.60% | |
| 7 | 60 kDa chaperonin OS=Borrelia miyamotoi GN=groL PE=3 SV=1 | AGT27591.1 | A0A1D8TE31\|A0A1D8TE31_9SPIR | 58966.8 | 100.00% | 2 | 2 | 3 | 0.32% | 22.10% | |
| 7 | Flagellin OS=*Borrelia miyamotoi* GN=AXH25_00725 PE=3 SV=1 | AGT27144.1 | A0A1D8TCQ1\|A0A1D8TCQ1_9SPIR | 35445.9 | 100.00% | 11 | 19 | 207 | 3.40% | 64.80% | |
| 7 | Ribosome-binding ATPase YchF OS=*Borrelia miyamotoi* GN=ychF PE=3 SV=1 | AGT27227.1 | A0A1D8TCW9\|A0A1D8TCW9_9SPIR | 40857.6 | 100.00% | 3 | 3 | 11 | 1.86% | 49.60% | |
| 7 | Variable large protein OS=Borrelia miyamotoi GN=AXH25_04660 PE=4 SV=1 | ALU64349.1 | A0A1D8TFY1\|A0A1D8TFY1_9SPIR | 35732.8 | 100.00% | 7 | 10 | 54 | 0.59% | 33.60% | |
| 7 | Variable large protein OS=*Borrelia miyamotoi* GN=AXH25_04665 PE=4 SV=1 | ALU64350.1 | A0A1D8TFG6\|A0A1D8TFG6_9SPIR | 36578.6 | 100.00% | 9 | 10 | 85 | 0.83% | 28.80% | |
| 7 | Variable large protein OS=*Borrelia miyamotoi* GN=AXH25_04680 PE=4 SV=1 | AOW96282.1 | A0A1D8TF92\|A0A1D8TF92_9SPIR | 35795.1 | 100.00% | 5 | 6 | 26 | 0.85% | 45.50% | |
| 7 | Variable large protein OS=Borrelia miyamotoi GN=AXH25_04900 PE=4 SV=1 | ALM31567.1 | A0A1D8TF85\|A0A1D8TF85_9SPIR | 36693 | 100.00% | 5 | 6 | 22 | 2.26% | 42.30% | |
| 7 | Variable large protein OS=*Borrelia miyamotoi* GN=vlpD10 PE=4 SV=1 | ALU64352.1 | A0A0X8ASK8\|A0A0X8ASK8_9SPIR | 36184 | 100.00% | 15 | 20 | 250 | 3.90% | 61.30% | |
| 8 | 60 kDa chaperonin OS=*Borrelia miyamotoi* GN=groL PE=3 SV=1 | AGT27591.1 | A0A1D8TE31\|A0A1D8TE31_9SPIR | 58966.8 | 100.00% | 4 | 5 | 11 | 0.20% | 14.30% | |
| 8 | Chaperone protein DnaK OS=Borrelia miyamotoi GN=dnaK PE=2 SV=1 | AGT27477.1 | A0A1D8TDM8\|A0A1D8TDM8_9SPIR | 68880.8 | 100.00% | 7 | 12 | 131 | 2.82% | 51.40% | |
| 8 | Flagellin OS=*Borrelia miyamotoi* GN=AXH25_00725 PE=3 SV=1 | AGT27144.1 | A0A1D8TCQ1\|A0A1D8TCQ1_9SPIR | 35445.9 | 100.00% | 12 | 27 | 579 | 0.60% | 35.10% | |
| 8 | Glycerophosphodiester phosphodiesterase OS=*Borrelia miyamotoi* GN=AXH25_01180 PE=4 SV=1 | AGT27237.1 | A0A1D8TD05\|A0A1D8TD05_9SPIR | 38703.2 | 100.00% | 9 | 10 | 38 | 0.74% | 22.20% | |
| 8 | Putative lipoprotein OS=*Borrelia miyamotoi* GN=AXH25_04705 PE=4 SV=1 | ALN43426.1 | A0A0U2RX32\|A0A0U2RX32_9SPIR | 35313.8 | 100.00% | 3 | 3 | 25 | 0.48% | 17.60% | |
| 8 | Uncharacterized protein OS=*Borrelia miyamotoi* GN=AXH25_01150 PE=4 SV=1 | WP_082002168.1 | A0A1D8TD18\|A0A1D8TD18_9SPIR | 29625.9 | 100.00% | 2 | 2 | 3 | 0.03% | 4.04% | |
| 8 | Uncharacterized protein OS=*Borrelia miyamotoi* GN=AXH25_03530 PE=4 SV=1 | AGT27657.1 | A0A1D8TE48\|A0A1D8TE48_9SPIR | 29675.2 | 100.00% | 5 | 5 | 17 | 2.31% | 41.90% | |
| 8 | Uncharacterized protein OS=*Borrelia miyamotoi* GN=AXH25_03690 PE=4 SV=1 | AGT27688.1 | A0A1D8TE76\|A0A1D8TE76_9SPIR | 77129.2 | 100.00% | 2 | 2 | 2 | 0.12% | 8.70% | |
| 9 | Flagellin OS=Borrelia miyamotoi GN=AXH25_00725 PE=3 SV=1 | AGT27144.1 | A0A1D8TCQ1\|A0A1D8TCQ1_9SPIR | 35445.9 | 100.00% | 10 | 19 | 271 | 0.60% | 20.20% | |
| 9 | Variable large protein OS=*Borrelia miyamotoi* GN=AXH25_04660 PE=4 SV=1 | ALU64349.1 | A0A1D8TFY1\|A0A1D8TFY1_9SPIR | 35732.8 | 100.00% | 5 | 5 | 58 | 0.95% | 39.00% | |
| 9 | Variable large protein OS=*Borrelia miyamotoi* GN=AXH25_04665 PE=4 SV=1 | ALU64350.1 | A0A1D8TFG6\|A0A1D8TFG6_9SPIR | 36578.6 | 100.00% | 18 | 25 | 343 | 0.29% | 33.00% | |
| 9 | Variable large protein OS=Borrelia miyamotoi GN=vlpD10 PE=4 SV=1 | ALU64352.1 | A0A0X8ASK8\|A0A0X8ASK8_9SPIR | 36184 | 100.00% | 4 | 5 | 21 | 0.25% | 30.50% | |
| 10 | ATP-dependent zinc metalloprotease FtsH OS=*Borrelia miyamotoi* OX=47466 GN=ftsH PE=3 SV=1 | AGT27731.1 | A0A1D8TEB8_9SPIR | 70973.2 | 100.00% | 2 | 2 | 10 | 2.79% | 55.10% | |
| 10 | Flagellin OS=*Borrelia miyamotoi* GN=AXH25_00725 PE=3 SV=1 | AGT27144.1 | A0A1D8TCQ1\|A0A1D8TCQ1_9SPIR | 35445.9 | 100.00% | 11 | 18 | 331 | 0.12% | 8.07% | |
| 10 | Serine protease OS=*Borrelia miyamotoi* GN=AXH25_00510 PE=4 SV=1 | WP_082002164.1 | A0A1D8TCL3\|A0A1D8TCL3_9SPIR | 52750.3 | 100.00% | 3 | 3 | 6 | 1.42% | 22.90% | |
| 10 | Variable large protein OS=*Borrelia miyamotoi* GN=AXH25_04665 PE=4 SV=1 | ALU64350.1 | A0A1D8TFG6\|A0A1D8TFG6_9SPIR | 36578.6 | 100.00% | 2 | 2 | 5 | 6.29% | 45.80% | |
| 10 | Variable large protein OS=*Borrelia miyamotoi* OX=47466 GN=vlpC2 PE=4 SV=1 | ALU64348.1 | A0A109PHQ5_9SPIR,  A0A1D8TFE5_9SPIR | 34513.6 | 100.00% | 28 | 35 | 3170 | 0.41% | 33.80% | |
| 10 | Variable small protein 1 OS=*Borrelia miyamotoi* GN=AXH25_04685 PE=4 SV=1 | AJA67245.2 | A0A1D8TFC5\|A0A1D8TFC5_9SPIR | 21956.2 | 100.00% | 3 | 3 | 14 | 0.27% | 16.10% | |
| 11 | ABC transporter ATP-binding protein OS=*Borrelia miyamotoi* OX=47466 GN=AXH25_03750 PE=4 SV=1 | AGT27698.1 | A0A1D8TE90_9SPIR | 34972.1 | 100.00% | 11 | 12 | 67 | 0.03% | 9.77% | |
| 11 | ATPase OS=*Borrelia miyamotoi* GN=AXH25_00845 PE=4 SV=1 | AGT27171.1 | A0A1D8TCR8\|A0A1D8TCR8_9SPIR | 36824.2 | 100.00% | 17 | 26 | 317 | 0.19% | 16.70% | |
| 11 | ATP-dependent zinc metalloprotease FtsH OS=*Borrelia miyamotoi* OX=47466 GN=ftsH PE=3 SV=1 | AGT27731.1 | A0A1D8TEB8_9SPIR | 70973.2 | 100.00% | 2 | 2 | 22 | 0.02% | 5.80% | |
| 11 | Carboxylesterase OS=*Borrelia miyamotoi* GN=AXH25_03185 PE=4 SV=1 | AGT27588.1 | A0A1D8TDY3\|A0A1D8TDY3_9SPIR | 37657.3 | 100.00% | 2 | 3 | 9 | 2.54% | 41.30% | |
| 11 | Flagellar protein OS=*Borrelia miyamotoi* GN=AXH25_03295 PE=4 SV=1 | AGT27611.1 | A0A1D8TE06\|A0A1D8TE06_9SPIR | 39285.2 | 100.00% | 2 | 2 | 2 | 0.54% | 10.70% | |
| 11 | Flagellin OS=*Borrelia miyamotoi* GN=AXH25_00725 PE=3 SV=1 | AGT27144.1 | A0A1D8TCQ1\|A0A1D8TCQ1_9SPIR | 35445.9 | 100.00% | 10 | 15 | 143 | 3.21% | 64.40% | |
| 11 | Glyceraldehyde-3-phosphate dehydrogenase OS=*Borrelia miyamotoi* GN=AXH25_00280 PE=3 SV=1 | AGT27060.1 | A0A1D8TCH5\|A0A1D8TCH5_9SPIR | 36305.1 | 100.00% | 17 | 22 | 225 | 0.20% | 24.40% | |
| 11 | Glycerophosphodiester phosphodiesterase OS=*Borrelia miyamotoi* GN=AXH25_01180 PE=4 SV=1 | AGT27237.1 | A0A1D8TD05\|A0A1D8TD05_9SPIR | 38703.2 | 100.00% | 11 | 12 | 65 | 0.07% | 4.88% | |
| 11 | Holliday junction ATP-dependent DNA helicase RuvB OS=*Borrelia miyamotoi* GN=ruvB PE=3 SV=1 | AGT27027.1 | A0A1D8TD15\|A0A1D8TD15_9SPIR | 38127.4 | 100.00% | 2 | 2 | 9 | 3.65% | 41.90% | |
| 11 | Protein HflC OS=*Borrelia miyamotoi* GN=AXH25_00980 PE=3 SV=1 | AGT27199.1 | A0A1D8TDG7\|A0A1D8TDG7_9SPIR | 37332.6 | 100.00% | 8 | 8 | 36 | 0.07% | 8.90% | |
| 11 | Protein HflK OS=*Borrelia miyamotoi* OX=47466 GN=AXH25_00975 PE=3 SV=1 | AGT27198.1 | A0A1D8TCY7_9SPIR,W5SJD0_9SPIR | 35333.5 | 100.00% | 8 | 9 | 50 | 0.06% | 13.10% | |
| 11 | Putative lipoprotein OS=*Borrelia miyamotoi* GN=AXH25_04705 PE=4 SV=1 | ALN43426.1 | A0A0U2RX32\|A0A0U2RX32_9SPIR | 35313.8 | 100.00% | 3 | 3 | 12 | 23.10% | 77.90% | |
| 11 | Rod shape-determining protein OS=*Borrelia miyamotoi* GN=AXH25_03545 PE=4 SV=1 | AGT27659.1 | A0A1D8TE68\|A0A1D8TE68_9SPIR | 38119.5 | 100.00% | 17 | 26 | 315 | 0.15% | 17.60% | |
| 11 | Spermidine/putrescine import ATP-binding protein PotA OS=*Borrelia miyamotoi* GN=potA PE=3 SV=1 | AGT27580.1 | A0A1D8TE04\|A0A1D8TE04_9SPIR | 39722.9 | 100.00% | 3 | 3 | 15 | 0.88% | 45.10% | |
| 11 | Threonylcarbamoyl-AMP synthase OS=*Borrelia miyamotoi* GN=AXH25_03645 PE=3 SV=1 | AGT27679.1 | A0A1D8TE63\|A0A1D8TE63_9SPIR | 37934.1 | 100.00% | 4 | 4 | 12 | 0.56% | 41.90% | |
| 11 | Uncharacterized protein OS=*Borrelia miyamotoi* GN=AXH25_00755 PE=4 SV=1 | AGT27150.2 | A0A1D8TCP6\|A0A1D8TCP6_9SPIR | 43946.6 | 100.00% | 3 | 3 | 5 | 3.39% | 63.60% | |
| 11 | Uncharacterized protein OS=*Borrelia miyamotoi* GN=AXH25_05900 PE=4 SV=1 | AOW96199.1 | A0A1D8TF03\|A0A1D8TF03_9SPIR | 37036.9 | 100.00% | 2 | 2 | 4 | 0.19% | 4.88% | |
| 11 | Uncharacterized protein OS=*Borrelia miyamotoi* OX=47466 GN=AXH25_02675 PE=4 SV=1 | AGT27497.1 | A0A1D8TDT4_9SPIR | 41742.6 | 100.00% | 2 | 2 | 8 | 0.10% | 7.65% | |
| 11 | Variable large protein OS=*Borrelia miyamotoi* OX=47466 GN=vlpC2 PE=4 SV=1 | ALU64348.1 | A0A109PHQ5_9SPIR,  A0A1D8TFE5_9SPIR | 34513.6 | 100.00% | 24 | 31 | 1642 | 0.02% | 6.05% | |
| 11 | Variable small protein 1 OS=*Borrelia miyamotoi* GN=AXH25_04685 PE=4 SV=1 | AJA67245.2 | A0A1D8TFC5\|A0A1D8TFC5_9SPIR | 21956.2 | 100.00% | 5 | 6 | 30 | 1.53% | 36.80% | |
| 12 | Uncharacterized protein OS=*Borrelia miyamotoi* OX=47466 GN=AXH25_03690 PE=4 SV=1 | AGT27688.1 | A0A1D8TE76_9SPIR | 77129.2 | 100.00% | 13 | 16 | 115 | 2.41% | 60.00% | |
| 13 | Uncharacterized protein OS=*Borrelia miyamotoi* OX=47466 GN=AXH25_02955 PE=4 SV=1 | AGT27550.1 | A0A1D8TDX3_9SPIR | 67447.4 | 100.00% | 16 | 18 | 162 | 0.70% | 39.50% | |
| 14 | Flagellar protein OS=*Borrelia miyamotoi* OX=47466 GN=AXH25_03295 PE=4 SV=1 | AGT27611.1 | A0A1D8TE06_9SPIR,  W5SCB3_9SPIR | 42328.2 | 100.00% | 3 | 3 | 5 | 0.10% | 7.06% | |
| 14 | Glyceraldehyde-3-phosphate dehydrogenase OS=*Borrelia miyamotoi* OX=47466 GN=AXH25_00280 PE=3 SV=1 | AGT27060.1 | A0A1D8TCH5_9SPIR | 36305.1 | 100.00% | 3 | 3 | 12 | 0.39% | 22.90% | |
| 14 | Variable large protein OS=*Borrelia miyamotoi* OX=47466 GN=AXH25_04655 PE=4 SV=1 | AOW96324.1 | A0A1D8TFE5_9SPIR | 35784.5 | 100.00% | 10 | 10 | 120 | 0.08% | 20.10% | |
| 15 | 50S ribosomal protein L25 OS=*Borrelia miyamotoi* OX=47466 GN=rplY PE=3 SV=1 | AGT27728.1 | A0A1D8TEA9_9SPIR | 20979.8 | 100.00% | 3 | 3 | 16 | 0.43% | 24.50% | |
| 15 | Variable small protein 1 OS=*Borrelia miyamotoi* OX=47466 GN=AXH25_06060 PE=4 SV=1 | AJA67245.2 | A0A1D8TF77_9SPIR,  A0A1D8TFC5_9SPIR | 21956.2 | 100.00% | 8 | 8 | 301 | 0.13% | 16.10% | |
| 16 | Peptidoglycan-binding protein LysM OS=*Borrelia miyamotoi* OX=47466 GN=AXH25_01585 PE=4 SV=1 | AGT27318.1 | A0A1D8TDR2_9SPIR | 43660.8 | 100.00% | 5 | 10 | 186 | 3.37% | 57.00% | |
| 16 | Variable large protein OS=*Borrelia miyamotoi* OX=47466 GN=AXH25_04655 PE=4 SV=1 | AOW96324.1 | A0A1D8TFE5_9SPIR | 35784.5 | 100.00% | 4 | 4 | 21 | 0.16% | 11.80% | |
| 16 | Variable small protein 1 OS=*Borrelia miyamotoi* OX=47466 GN=AXH25_06060 PE=4 SV=1 | AJA67245.2 | A0A1D8TF77_9SPIR,  A0A1D8TFC5_9SPIR | 21956.2 | 100.00% | 4 | 4 | 53 | 0.13% | 14.70% | |
| 17 | Variable small protein 1 OS=*Borrelia miyamotoi* OX=47466 GN=AXH25_06060 PE=4 SV=1 | AJA67245.2 | A0A1D8TF77_9SPIR,  A0A1D8TFC5_9SPIR | 21956.2 | 100.00% | 4 | 4 | 77 | 0.05% | 9.02% | |
| 18 | ABC transporter substrate-binding protein OS=*Borrelia miyamotoi* OX=47466 GN=AXH25_01610 PE=4 SV=1 | AGT27323.1 | A0A1D8TD91_9SPIR | 60830.7 | 100.00% | 12 | 14 | 189 | 0.04% | 8.36% | |
| 18 | ABC transporter substrate-binding protein OS=*Borrelia miyamotoi* OX=47466 GN=AXH25_01615 PE=4 SV=1 | AGT27324.1 | A0A1D8TD60_9SPIR | 61010.9 | 100.00% | 19 | 22 | 186 | 0.07% | 6.45% | |
| 18 | Lysine--tRNA ligase OS=*Borrelia miyamotoi* OX=47466 GN=lysS PE=3 SV=1 | AGT27602.1 | A0A1D8TEK4_9SPIR | 60631.4 | 100.00% | 8 | 9 | 51 | 14.20% | 76.50% | |
| 18 | Ribonuclease Y OS=*Borrelia miyamotoi* OX=47466 GN=rny PE=3 SV=1 | AGT27464.1 | A0A1D8TDK9_9SPIR,  W5SDA3_9SPIR | 58398.3 | 100.00% | 3 | 3 | 7 | 6.39% | 50.90% | |
| 18 | Transcription termination/antitermination protein NusA OS=*Borrelia miyamotoi* OX=47466 GN=nusA PE=3 SV=1 | AGT27741.1 | A0A1D8TEC3_9SPIR | 54661.7 | 100.00% | 2 | 2 | 3 | 0.32% | 23.30% | |
| 19 | 60 kDa chaperonin OS=*Borrelia miyamotoi* OX=47466 GN=groL PE=3 SV=1 | AGT27591.1 | A0A1D8TE31_9SPIR | 58966.8 | 100.00% | 3 | 3 | 6 | 0.46% | 8.93% | |
| 19 | ABC transporter substrate-binding protein OS=*Borrelia miyamotoi* OX=47466 GN=AXH25_01610 PE=4 SV=1 | AGT27323.1 | A0A1D8TD91_9SPIR | 60830.7 | 100.00% | 4 | 4 | 37 | 0.88% | 8.78% | |
| 20 | ABC transporter ATP-binding protein OS=*Borrelia miyamotoi* OX=47466 GN=AXH25_03750 PE=4 SV=1 | AGT27698.1 | A0A1D8TE90_9SPIR | 34972.1 | 100.00% | 2 | 2 | 10 | 1.30% | 21.90% | |
| 20 | Glyceraldehyde-3-phosphate dehydrogenase OS=*Borrelia miyamotoi* OX=47466 GN=AXH25_00280 PE=3 SV=1 | AGT27060.1 | A0A1D8TCH5_9SPIR | 36305.1 | 100.00% | 16 | 26 | 549 | 0.60% | 10.60% | |
| 20 | Uncharacterized protein OS=*Borrelia miyamotoi* OX=47466 GN=AXH25_00755 PE=4 SV=1 | AGT27150.2 | A0A1D8TCP6_9SPIR | 43946.6 | 100.00% | 4 | 4 | 17 | 2.27% | 26.20% | |
| 20 | Uncharacterized protein OS=*Borrelia miyamotoi* OX=47466 GN=AXH25_02675 PE=4 SV=1 | AGT27497.1 | A0A1D8TDT4_9SPIR | 41742.6 | 100.00% | 2 | 2 | 4 | 0.05% | 8.65% | |
| 20 | Variable large protein OS=*Borrelia miyamotoi* OX=47466 GN=AXH25_04655 PE=4 SV=1 | AOW96324.1 | A0A1D8TFE5_9SPIR | 35784.5 | 100.00% | 7 | 7 | 53 | 0.12% | 12.20% | |
| 20 | Variable small protein 1 OS=*Borrelia miyamotoi* OX=47466 GN=AXH25_06060 PE=4 SV=1 | AJA67245.2 | A0A1D8TF77_9SPIR,  A0A1D8TFC5_9SPIR | 21956.2 | 100.00% | 2 | 2 | 8 | 1.24% | 39.20% | |
| 21 | Chaperone protein DnaK OS=*Borrelia miyamotoi* GN=dnaK PE=2 SV=1 | AGT27477.1 | A0A1D8TDM8\|A0A1D8TDM8_9SPIR | 68880.8 | 100.00% | 40 | 80 | 1908 | 0.18% | 14.80% | |
| 21 | Flagellin OS=*Borrelia miyamotoi* GN=AXH25_00725 PE=3 SV=1 | AGT27144.1 | A0A1D8TCQ1\|A0A1D8TCQ1_9SPIR | 35445.9 | 100.00% | 10 | 16 | 209 | 3.33% | 35.10% | |
| 21 | Uncharacterized protein OS=*Borrelia miyamotoi* GN=AXH25_05530 PE=4 SV=1 | AOW96138.1 | A0A1D8TES8\|A0A1D8TES8_9SPIR,  A0A1D8TEX7\|A0A1D8TEX7_9SPIR,  A0A1D8TEY7\|A0A1D8TEY7_9SPIR | 21973.7 | 100.00% | 3 | 3 | 13 | 2.01% | 16.60% | |
| 22 | 60 kDa chaperonin OS=*Borrelia miyamotoi* GN=groL PE=3 SV=1 | AGT27591.1 | A0A1D8TE31\|A0A1D8TE31_9SPIR | 58966.8 | 100.00% | 8 | 13 | 112 | 0.23% | 15.50% | |
| 22 | ABC transporter ATP-binding protein OS=*Borrelia miyamotoi* OX=47466 GN=AXH25_03750 PE=4 SV=1 | AGT27698.1 | A0A1D8TE90_9SPIR | 34972.1 | 100.00% | 2 | 2 | 4 | 0.57% | 20.10% | |
| 22 | DNA-directed RNA polymerase subunit alpha OS=*Borrelia miyamotoi* GN=rpoA PE=3 SV=1 | AGT27462.1 | A0A1D8TDR4\|A0A1D8TDR4_9SPIR | 38142.3 | 100.00% | 18 | 28 | 483 | 0.92% | 20.10% | |
| 22 | Enolase OS=*Borrelia miyamotoi* GN=eno PE=3 SV=1 | AGT27330.1 | A0A1D8TDA6\|A0A1D8TDA6_9SPIR | 47094.7 | 100.00% | 2 | 2 | 4 | 1.76% | 26.10% | |
| 22 | Flagellar assembly protein FliH OS=*Borrelia miyamotoi* GN=AXH25_01425 PE=4 SV=1 | AGT27286.1 | A0A1D8TD19\|A0A1D8TD19_9SPIR | 35109.5 | 100.00% | 3 | 3 | 11 | 1.73% | 34.00% | |
| 22 | Flagellin OS=*Borrelia miyamotoi* GN=AXH25_00725 PE=3 SV=1 | AGT27144.1 | A0A1D8TCQ1\|A0A1D8TCQ1_9SPIR | 35445.9 | 100.00% | 13 | 31 | 1063 | 0.48% | 17.10% | |
| 22 | Glycerophosphodiester phosphodiesterase OS=*Borrelia miyamotoi* GN=AXH25_01180 PE=4 SV=1 | AGT27237.1 | A0A1D8TD05\|A0A1D8TD05_9SPIR | 38703.2 | 100.00% | 15 | 17 | 114 | 0.07% | 6.43% | |
| 22 | Putative lipoprotein OS=*Borrelia miyamotoi* GN=AXH25_04705 PE=4 SV=1 | ALN43426.1 | A0A0U2RX32\|A0A0U2RX32_9SPIR | 35313.8 | 100.00% | 13 | 20 | 218 | 0.03% | 4.79% | |
| 22 | Spermidine/putrescine ABC transporter substrate-binding protein OS=*Borrelia miyamotoi* OX=47466 GN=AXH25_03150 PE=4 SV=1 | AGT27580.1 | A0A1D8TE21_9SPIR | 40506.7 | 100.00% | 13 | 21 | 340 | 0.08% | 5.50% | |
| 22 | Uncharacterized protein OS=*Borrelia miyamotoi* GN=AXH25_04935 PE=4 SV=1 | AOW96267.1 | A0A1D8TF49\|A0A1D8TF49_9SPIR | 35497.6 | 100.00% | 2 | 3 | 12 | 0.52% | 7.37% | |
| 22 | Variable large protein OS=*Borrelia miyamotoi* GN=AXH25_04660 PE=4 SV=1 | ALU64349.1 | A0A1D8TFY1\|A0A1D8TFY1_9SPIR | 35732.8 | 100.00% | 16 | 19 | 237 | 0.12% | 6.49% | |
| 22 | Variable large protein OS=*Borrelia miyamotoi* GN=AXH25_04665 PE=4 SV=1 | ALU64350.1 | A0A1D8TFG6\|A0A1D8TFG6_9SPIR | 36578.6 | 100.00% | 2 | 2 | 22 | 6.43% | 53.40% | |
| 22 | Variable large protein OS=*Borrelia miyamotoi* GN=AXH25_04680 PE=4 SV=1 | AOW96282.1 | A0A1D8TF92\|A0A1D8TF92_9SPIR | 35795.1 | 100.00% | 3 | 3 | 6 | 0.20% | 11.90% | |
| 22 | Variable large protein OS=*Borrelia miyamotoi* GN=AXH25_04900 PE=4 SV=1 | ALM31567.1 | A0A1D8TF85\|A0A1D8TF85_9SPIR | 36693 | 100.00% | 12 | 17 | 217 | 0.05% | 8.06% | |
| 22 | Variable large protein OS=*Borrelia miyamotoi* GN=AXH25_06025 PE=4 SV=1 | ALM31565.1 | A0A1D8TF59\|A0A1D8TF59_9SPIR | 34299 | 100.00% | 8 | 9 | 42 | 0.62% | 25.10% | |
| 22 | Variable large protein OS=*Borrelia miyamotoi* GN=vlpD10 PE=4 SV=1 | ALU64352.1 | A0A0X8ASK8\|A0A0X8ASK8_9SPIR | 36184 | 100.00% | 2 | 3 | 29 | 0.09% | 10.80% | |
| 22 | Variable large protein OS=*Borrelia miyamotoi* OX=47466 GN=AXH25_04820 PE=4 SV=1 | AOW96302.1 | A0A1D8TFA6_9SPIR | 34262.8 | 100.00% | 5 | 6 | 23 | 16.50% | 71.80% | |
| 22 | Variable large protein OS=*Borrelia miyamotoi* OX=47466 GN=vlpC2 PE=4 SV=1 | ALU64348.1 | A0A109PHQ5_9SPIR,  A0A1D8TFE5_9SPIR | 34513.6 | 100.00% | 14 | 15 | 320 | 1.81% | 41.30% | |
| 23 | Uncharacterized protein OS=*Borrelia miyamotoi* GN=AXH25_01115 PE=4 SV=1 | AGT27223.1 | A0A1D8TCX1_9SPIR | 15128.1 | 100.00% | 5 | 7 | 80 | 0.11% | 10.80% | |
| 23 | Flagellar hook assembly protein FlgD OS=*Borrelia miyamotoi* OX=47466 GN=AXH25_01400 PE=4 SV=1 | AGT27281.1 | A0A1D8TD23_9SPIR | 15664.8 | 100.00% | 2 | 2 | 6 | 0.99% | 23.30% | |
| 23 | 30S ribosomal protein S10 OS=*Borrelia miyamotoi* OX=47466 GN=rpsJ PE=3 SV=1 | AGT27452.1 | A0A1D8TDN1_9SPIR,  W5SCV5_9SPIR | 11849.8 | 100.00% | 2 | 2 | 3 | 0.04% | 7.14% | |
| 23 | Heat-shock protein OS=*Borrelia miyamotoi* GN=AXH25_02180 PE=3 SV=1 | WP_043867868.1 | A0A1D8TEJ4_9SPIR | 16753.7 | 100.00% | 8 | 13 | 150 | 4.28% | 59.80% | |
| 23 | DUF1640 domain-containing protein OS=*Borrelia miyamotoi* GN=AXH25_04770 PE=4 SV=1 | AOW96294.1 | A0A1D8TFB5_9SPIR | 17899.2 | 100.00% | 3 | 3 | 13 | 0.04% | 6.03% | |
| 23 | Uncharacterized protein OS=*Borrelia miyamotoi* OX=47466 GN=AXH25_04455 PE=4 SV=1 | AOW96390.1 | A0A1D8TFI1_9SPIR | 18895.8 | 100.00% | 5 | 5 | 31 | 0.10% | 12.10% | |
| 23 | Uncharacterized protein OS=*Borrelia miyamotoi* GN=AXH25_04480 PE=4 SV=1 | AOW96394.1 | A0A1D8TFM3_9SPIR | 17007.6 | 100.00% | 8 | 15 | 517 | 9.41% | 45.80% | |
| 24 | Elongation factor G OS=*Borrelia miyamotoi* GN=fusA PE=3 SV=1 | AGT27495.1 | A0A1D8TDP5\|A0A1D8TDP5_9SPIR | 77150.6 | 100.00% | 2 | 2 | 2 | 1.01% | 56.90% | |
| 24 | Uncharacterized protein OS=*Borrelia miyamotoi* OX=47466 GN=AXH25_04480 PE=4 SV=1 | AOW96394.1 | A0A1D8TFM3_9SPIR | 17007.6 | 100.00% | 10 | 18 | 3051 | 1.93% | 44.40% | |
| 25 | 30S ribosomal protein S10 OS=*Borrelia miyamotoi* OX=47466 GN=rpsJ PE=3 SV=1 | AGT27452.1 | A0A1D8TDN1_9SPIR,  W5SCV5_9SPIR | 11849.8 | 100.00% | 2 | 2 | 7 | 3.23% | 41.70% | |
| 25 | 50S ribosomal protein L21 OS=*Borrelia miyamotoi* OX=47466 GN=rplU PE=3 SV=1 | AGT27721.1 | A0A1D8TEA7_9SPIR | 11990.7 | 100.00% | 4 | 7 | 51 | 0.11% | 38.10% | |
| 25 | Uncharacterized protein OS=*Borrelia miyamotoi* OX=47466 GN=AXH25_04480 PE=4 SV=1 | AOW96394.1 | A0A1D8TFM3_9SPIR | 17007.6 | 100.00% | 6 | 9 | 401 | 2.10% | 48.70% | |
| 25 | Variable small protein 1 OS=*Borrelia miyamotoi* GN=AXH25_04685 PE=4 SV=1 | AJA67245.2 | A0A1D8TFC5\|A0A1D8TFC5_9SPIR | 21956.2 | 100.00% | 11 | 14 | 358 | 0.20% | 16.20% | |
| 25 | Uncharacterized protein OS=Borrelia miyamotoi GN=AXH25_04350 PE=4 SV=1 | AOW96369.1 | A0A1D8TFG9\|A0A1D8TFG9_9SPIR | 28718.6 | 100.00% | 3 | 4 | 15 | 0.13% | 15.60% | |
| 26 | 50S ribosomal protein L5 OS=*Borrelia miyamotoi* OX=47466 GN=rplE PE=3 SV=1 | AGT26990.1 | A0A1D8TDI9_9SPIR,  W5SIL4_9SPIR | 20315.7 | 100.00% | 2 | 2 | 6 | 0.05% | 25.20% | |
| 26 | 50S ribosomal protein L9 OS=*Borrelia miyamotoi* OX=47466 GN=rplI PE=3 SV=1 | AGT27115.1 | A0A1D8TD98_9SPIR | 19103.6 | 100.00% | 4 | 4 | 27 | 1.92% | 52.90% | |
| 26 | Variable small protein 1 OS=*Borrelia miyamotoi* OX=47466 GN=AXH25_06060 PE=4 SV=1 | AJA67245.2 | A0A1D8TF77_9SPIR,  A0A1D8TFC5_9SPIR | 21956.2 | 100.00% | 3 | 3 | 29 | 0.37% | 39.60% | |
| 27 | 50S ribosomal protein L5 OS=*Borrelia miyamotoi* OX=47466 GN=rplE PE=3 SV=1 | AGT26990.1 | A0A1D8TDI9_9SPIR,  W5SIL4_9SPIR | 20315.7 | 100.00% | 4 | 5 | 18 | 0.26% | 12.80% | |
| 27 | 50S ribosomal protein L9 OS=*Borrelia miyamotoi* OX=47466 GN=rplI PE=3 SV=1 | AGT27115.1 | A0A1D8TD98_9SPIR | 19103.6 | 100.00% | 4 | 4 | 36 | 0.22% | 35.30% | |
| 27 | Variable small protein 1 OS=*Borrelia miyamotoi* OX=47466 GN=AXH25_06060 PE=4 SV=1 | AJA67245.2 | A0A1D8TF77_9SPIR,  A0A1D8TFC5_9SPIR | 21956.2 | 100.00% | 4 | 4 | 74 | 3.04% | 51.20% | |
| 28 | Flagellar protein OS=*Borrelia miyamotoi* OX=47466 GN=AXH25_01410 PE=4 SV=1 | AGT27283.1 | A0A1D8TD71_9SPIR,  W5SEP2_9SPIR | 24133.2 | 100.00% | 2 | 2 | 24 | 0.97% | 45.90% | |
| 28 | Transcription termination/antitermination protein NusG OS=*Borrelia miyamotoi* OX=47466 GN=nusG PE=3 SV=1 | AGT27376.1 | A0A1D8TDF8_9SPIR | 21178.2 | 100.00% | 12 | 18 | 469 | 0.06% | 17.00% | |
| 28 | V-type proton ATPase subunit E OS=*Borrelia miyamotoi* OX=47466 GN=atpE PE=3 SV=1 | AGT27099.1 | A0A1D8TCL5_9SPIR | 23031.6 | 100.00% | 10 | 13 | 370 | 0.03% | 27.20% | |
| 29 | Transcription termination/antitermination protein NusG OS=*Borrelia miyamotoi* OX=47466 GN=nusG PE=3 SV=1 | AGT27376.1 | A0A1D8TDF8_9SPIR | 21178.2 | 100.00% | 6 | 8 | 65 | 1.82% | 56.70% | |
| 29 | Uncharacterized protein OS=*Borrelia miyamotoi* OX=47466 GN=AXH25_03275 PE=4 SV=1 | AGT27607.1 | A0A1D8TE05_9SPIR | 24945.6 | 100.00% | 5 | 8 | 123 | 0.16% | 20.60% | |
| 29 | V-type proton ATPase subunit E OS=*Borrelia miyamotoi* OX=47466 GN=atpE PE=3 SV=1 | AGT27099.1 | A0A1D8TCL5_9SPIR | 23031.6 | 100.00% | 10 | 12 | 367 | 0.13% | 14.80% | |
| 30 | 50S ribosomal protein L25 OS=*Borrelia miyamotoi* GN=rplY PE=3 SV=1 | AGT27728.1 | A0A1D8TEA9\|A0A1D8TEA9_9SPIR | 20979.8 | 100.00% | 2 | 2 | 11 | 0.22% | 26.50% | |
| 30 | Flagellin OS=*Borrelia miyamotoi* GN=AXH25_00725 PE=3 SV=1 | AGT27144.1 | A0A1D8TCQ1\|A0A1D8TCQ1_9SPIR | 35445.9 | 100.00% | 11 | 24 | 764 | 0.33% | 35.80% | |
| 30 | Peptidoglycan-binding protein LysM OS=*Borrelia miyamotoi* GN=AXH25_01585 PE=4 SV=1 | AGT27318.1 | A0A1D8TDR2\|A0A1D8TDR2_9SPIR | 43660.8 | 100.00% | 2 | 2 | 6 | 6.26% | 62.60% | |
| 30 | Uncharacterized protein OS=*Borrelia miyamotoi* GN=AXH25_01095 PE=4 SV=1 | AGT27219.1 | A0A1D8TD07\|A0A1D8TD07_9SPIR | 28447.9 | 100.00% | 4 | 4 | 11 | 0.81% | 43.20% | |
| 30 | Variable small protein 1 OS=*Borrelia miyamotoi* GN=AXH25_04685 PE=4 SV=1 | AJA67245.2 | A0A1D8TFC5\|A0A1D8TFC5_9SPIR | 21956.2 | 100.00% | 20 | 27 | 1291 | 0.03% | 6.94% | |
| 31 | Phosphate ABC transporter substrate-binding protein OS=*Borrelia miyamotoi* GN=AXH25_01045 PE=4 SV=1 | AGT27210.1 | A0A1D8TDH6\|A0A1D8TDH6_9SPIR | 31380.9 | 100.00% | 13 | 26 | 1118 | 24.30% | 67.70% | |
| 31 | Phosphate import ATP-binding protein PstB OS=*Borrelia miyamotoi* GN=pstB PE=3 SV=1 | AGT27213.1 | A0A1D8TCW2\|A0A1D8TCW2_9SPIR | 28693.2 | 100.00% | 9 | 9 | 53 | 0.07% | 18.40% | |
| 31 | Triosephosphate isomerase OS=*Borrelia miyamotoi* GN=tpiA PE=3 SV=1 | AGT27058.1 | A0A1D8TD53\|A0A1D8TD53_9SPIR | 27818.6 | 100.00% | 13 | 18 | 146 | 0.49% | 40.40% | |
| 31 | Uncharacterized protein OS=*Borrelia miyamotoi* GN=AXH25_01150 PE=4 SV=1 | WP_082002168.1 | A0A1D8TD18\|A0A1D8TD18_9SPIR | 29625.9 | 100.00% | 4 | 4 | 13 | 3.84% | 44.50% | |
| 31 | Variable large protein OS=*Borrelia miyamotoi* OX=47466 GN=vlpC2 PE=4 SV=1 | ALU64348.1 | A0A109PHQ5_9SPIR,  A0A1D8TFE5_9SPIR | 34513.6 | 100.00% | 5 | 5 | 59 | 3.37% | 44.30% | |
| 31 | Variable small protein 1 OS=*Borrelia miyamotoi* GN=AXH25_04685 PE=4 SV=1 | AJA67245.2 | A0A1D8TFC5\|A0A1D8TFC5_9SPIR | 21956.2 | 100.00% | 5 | 5 | 15 | 0.06% | 11.50% | |
| 32 | 50S ribosomal protein L25 OS=*Borrelia miyamotoi* GN=rplY PE=3 SV=1 | AGT27728.1 | A0A1D8TEA9\|A0A1D8TEA9_9SPIR | 20979.8 | 100.00% | 6 | 6 | 21 | 0.27% | 21.30% | |
| 32 | 50S ribosomal protein L3 OS=*Borrelia miyamotoi* OX=47466 GN=rplC PE=3 SV=1 | AGT26984.1 | A0A1D8TE50_9SPIR,W5SE62_9SPIR | 22686.9 | 100.00% | 2 | 2 | 2 | 0.29% | 16.00% | |
| 32 | 5'-methylthioadenosine/S-adenosylhomocysteine nucleosidase OS=*Borrelia miyamotoi* GN=AXH25_04575 PE=3 SV=1 | WP_082583095.1 | A0A1D8TFZ2\|A0A1D8TFZ2_9SPIR | 25403.4 | 100.00% | 5 | 6 | 103 | 0.18% | 29.10% | |
| 32 | Flagellin OS=*Borrelia miyamotoi* GN=AXH25_00725 PE=3 SV=1 | AGT27144.1 | A0A1D8TCQ1\|A0A1D8TCQ1_9SPIR | 35445.9 | 100.00% | 10 | 21 | 361 | 0.37% | 21.30% | |
| 32 | Protein GrpE OS=*Borrelia miyamotoi* GN=grpE PE=3 SV=1 | AGT27478.1 | A0A1D8TDR8\|A0A1D8TDR8_9SPIR | 21279.3 | 100.00% | 4 | 4 | 31 | 0.75% | 20.10% | |
| 32 | Uncharacterized protein OS=*Borrelia miyamotoi* FR64b OX=1292392 GN=BOM_0406 PE=4 SV=1 | AGT27434.1 | W5SCX4_9SPIR | 27162.4 | 100.00% | 2 | 2 | 16 | 0.21% | 10.80% | |
| 32 | Variable large protein OS=*Borrelia miyamotoi* OX=47466 GN=vlpC2 PE=4 SV=1 | ALU64348.1 | A0A109PHQ5_9SPIR,  A0A1D8TFE5_9SPIR | 34513.6 | 100.00% | 9 | 10 | 132 | 4.06% | 81.00% | |
| 32 | Variable small protein 1 OS=*Borrelia miyamotoi* GN=AXH25_04685 PE=4 SV=1 | AJA67245.2 | A0A1D8TFC5\|A0A1D8TFC5_9SPIR | 21956.2 | 100.00% | 20 | 31 | 2337 | 3.21% | 60.20% | |
| 33 | DNA-directed RNA polymerase subunit alpha OS=*Borrelia miyamotoi* OX=47466 GN=rpoA PE=3 SV=1 | AGT27462.1 | A0A1D8TDR4_9SPIR,  W5SCT1_9SPIR | 38169.2 | 100.00% | 3 | 3 | 8 | 0.66% | 44.60% | |
| 33 | Flagellar assembly protein FliH OS=*Borrelia miyamotoi* OX=47466 GN=AXH25_01425 PE=4 SV=1 | AGT27286.1 | A0A1D8TD19_9SPIR | 35109.5 | 100.00% | 3 | 3 | 10 | 1.25% | 28.70% | |
| 33 | Flagellin OS=*Borrelia miyamotoi* OX=47466 GN=AXH25_00725 PE=3 SV=1 | AGT27144.1 | A0A1D8TCQ1_9SPIR | 35445.9 | 100.00% | 12 | 25 | 653 | 3.74% | 52.70% | |
| 33 | Glyceraldehyde-3-phosphate dehydrogenase OS=*Borrelia miyamotoi* OX=47466 GN=AXH25_00280 PE=3 SV=1 | AGT27060.1 | A0A1D8TCH5_9SPIR | 36305.1 | 100.00% | 2 | 2 | 6 | 0.10% | 15.30% | |
| 33 | Variable large protein OS=*Borrelia miyamotoi* OX=47466 GN=AXH25_04655 PE=4 SV=1 | AOW96324.1 | A0A1D8TFE5_9SPIR | 35784.5 | 100.00% | 14 | 17 | 457 | 7.05% | 39.80% | |
| 33 | Variable large protein OS=*Borrelia miyamotoi* OX=47466 GN=AXH25_04660 PE=4 SV=1 | ALU64349.1 | A0A1D8TFY1_9SPIR | 35732.8 | 100.00% | 7 | 7 | 47 | 0.06% | 6.43% | |
| 33 | Variable large protein OS=*Borrelia miyamotoi* OX=47466 GN=AXH25_04900 PE=4 SV=1 | ALM31567.1 | A0A1D8TF85_9SPIR | 36693 | 100.00% | 4 | 4 | 16 | 0.10% | 16.00% | |
| 34 | Variable small protein 1 OS=*Borrelia miyamotoi* OX=47466 GN=AXH25_06060 PE=4 SV=1 | AJA67245.2 | A0A1D8TF77_9SPIR,  A0A1D8TFC5_9SPIR | 21956.2 | 100.00% | 6 | 6 | 174 | 11.90% | 72.90% | |
| 35 | 50S ribosomal protein L9 OS=*Borrelia miyamotoi* OX=47466 GN=rplI PE=3 SV=1 | AGT27115.1 | A0A1D8TD98_9SPIR | 19103.6 | 100.00% | 2 | 2 | 4 | 11.70% | 61.50% | |
| 35 | Uncharacterized protein OS=*Borrelia miyamotoi* OX=47466 GN=AXH25_03865 PE=4 SV=1 | AGT27719.1 | A0A1D8TEA5_9SPIR | 20199.6 | 100.00% | 3 | 4 | 78 | 0.56% | 41.30% | |
| 35 | Variable small protein 1 OS=*Borrelia miyamotoi* OX=47466 GN=AXH25_06060 PE=4 SV=1 | AJA67245.2 | A0A1D8TF77_9SPIR,  A0A1D8TFC5_9SPIR | 21956.2 | 100.00% | 2 | 2 | 8 | 1.53% | 65.40% | |
| 36 | Peptidoglycan-binding protein LysM OS=*Borrelia miyamotoi* OX=47466 GN=AXH25_01585 PE=4 SV=1 | AGT27318.1 | A0A1D8TDR2_9SPIR | 43660.8 | 100.00% | 2 | 2 | 9 | 0.14% | 23.00% | |
| 36 | Variable small protein 1 OS=*Borrelia miyamotoi* OX=47466 GN=AXH25_06060 PE=4 SV=1 | AJA67245.2 | A0A1D8TF77_9SPIR,  A0A1D8TFC5_9SPIR | 21956.2 | 100.00% | 11 | 13 | 415 | 0.61% | 20.30% | |
| 37 | 50S ribosomal protein L19 OS=*Borrelia miyamotoi* FR64b OX=1292392 GN=rplS PE=3 SV=1 | AGT27642.1 | A0A1D8TE22_9SPIR,  W5SHY1_9SPIR | 16592 | 100.00% | 2 | 2 | 5 | 0.16% | 23.30% | |
| 37 | Probable chemoreceptor glutamine deamidase CheD OS=*Borrelia miyamotoi* OX=47466 GN=cheD PE=3 SV=1 | AGT27553.1 | A0A1D8TDV7_9SPIR | 17968.5 | 100.00% | 3 | 3 | 12 | 0.18% | 38.30% | |
| 37 | Uncharacterized protein OS=*Borrelia miyamotoi* OX=47466 GN=AXH25_03690 PE=4 SV=1 | AGT27688.1 | A0A1D8TE76_9SPIR | 77129.2 | 100.00% | 2 | 2 | 20 | 0.02% | 11.00% | |
| 38 | 30S ribosomal protein S7 OS=*Borrelia miyamotoi* OX=47466 GN=rpsG PE=3 SV=1 | AGT26980.1 | A0A1D8TDB2_9SPIR,  W5SIV7_9SPIR | 18159 | 100.00% | 2 | 2 | 9 | 0.86% | 37.70% | |
| 38 | Flagellar basal-body rod protein FlgC OS=*Borrelia miyamotoi* OX=47466 GN=AXH25_01445 PE=3 SV=1 | AGT27290.1 | A0A1D8TD31_9SPIR,  W5SJ39_9SPIR | 16635.4 | 100.00% | 2 | 2 | 6 | 0.11% | 17.70% | |
| 38 | Probable chemoreceptor glutamine deamidase CheD OS=*Borrelia miyamotoi* OX=47466 GN=cheD PE=3 SV=1 | AGT27553.1 | A0A1D8TDV7_9SPIR | 17968.5 | 100.00% | 4 | 4 | 34 | 3.00% | 39.80% | |
| 38 | Variable large protein OS=*Borrelia miyamotoi* OX=47466 GN=AXH25_04655 PE=4 SV=1 | AOW96324.1 | A0A1D8TFE5_9SPIR | 35784.5 | 100.00% | 2 | 2 | 10 | 0.26% | 28.00% | |
| 39 | ATP-dependent Clp protease ATP-binding subunit ClpX OS=*Borrelia miyamotoi* OX=47466 GN=clpX PE=3 SV=1 | AGT27557.1 | A0A1D8TDX7_9SPIR | 48053.7 | 100.00% | 2 | 2 | 2 | 0.18% | 10.00% | |
| 39 | Inosine-5'-monophosphate dehydrogenase OS=*Borrelia miyamotoi* OX=47466 GN=guaB PE=3 SV=1 | AJA67229.1 | A0A1D8TFB0_9SPIR | 52906.8 | 100.00% | 7 | 7 | 38 | 1.50% | 44.80% | |
| 39 | M18 family aminopeptidase OS=*Borrelia miyamotoi* OX=47466 GN=AXH25_01770 PE=3 SV=1 | AGT27354.1 | A0A1D8TDB6_9SPIR | 51863.9 | 100.00% | 7 | 7 | 44 | 19.40% | 73.80% | |
| 39 | Pyruvate kinase OS=*Borrelia miyamotoi* OX=47466 GN=AXH25_01700 PE=3 SV=1 | AGT27339.1 | A0A1D8TDB7_9SPIR | 53091.4 | 100.00% | 21 | 29 | 323 | 1.03% | 66.20% | |
| 39 | Uncharacterized protein OS=*Borrelia miyamotoi* OX=47466 GN=AXH25_00190 PE=4 SV=1 | AGT27044.1 | A0A1D8TCF5_9SPIR | 58788.6 | 100.00% | 3 | 3 | 7 | 0.09% | 8.16% | |
| 39 | Variable large protein OS=*Borrelia miyamotoi* OX=47466 GN=vlpC2 PE=4 SV=1 | ALU64348.1 | A0A109PHQ5_9SPIR,  A0A1D8TFE5_9SPIR | 34513.6 | 100.00% | 4 | 4 | 23 | 0.11% | 10.80% | |
| 40 | Flagellar motor switch protein FliM OS=*Borrelia miyamotoi* OX=47466 GN=AXH25_01370 PE=3 SV=1 | AGT27275.1 | A0A1D8TD54_9SPIR,  W5SJ56_9SPIR | 39357.5 | 100.00% | 2 | 2 | 4 | 7.48% | 41.90% | |
| 40 | Variable large protein OS=*Borrelia miyamotoi* OX=47466 GN=vlpC2 PE=4 SV=1 | ALU64348.1 | A0A109PHQ5_9SPIR,  A0A1D8TFE5_9SPIR | 34513.6 | 100.00% | 4 | 4 | 24 | 3.03% | 37.10% | |
|  | | | | | | | | | | |  |

**Supplemental Figure 1.** Visualization of purified recombinant *B. miyamotoi* proteins. Approximately 100 ng of individual recombinant protein or borrelial whole cell lysate was separated via SDS-PAGE and subsequently stained with Coomassie (a) or tagged with anti-His (b). Molecular weight of proteins is expressed in kDa. P.l. = putative lipoprotein; U.p. = uncharacterized protein; Vlp = variable large protein; Vsp = variable small protein; GlpQ = glycerophosphodiester phosphodiesterase; *B. m.* lysate = *B. miyamotoi* whole cell lysate*; B. b.* lysate = *B. burgdorferi* whole cell lysate.


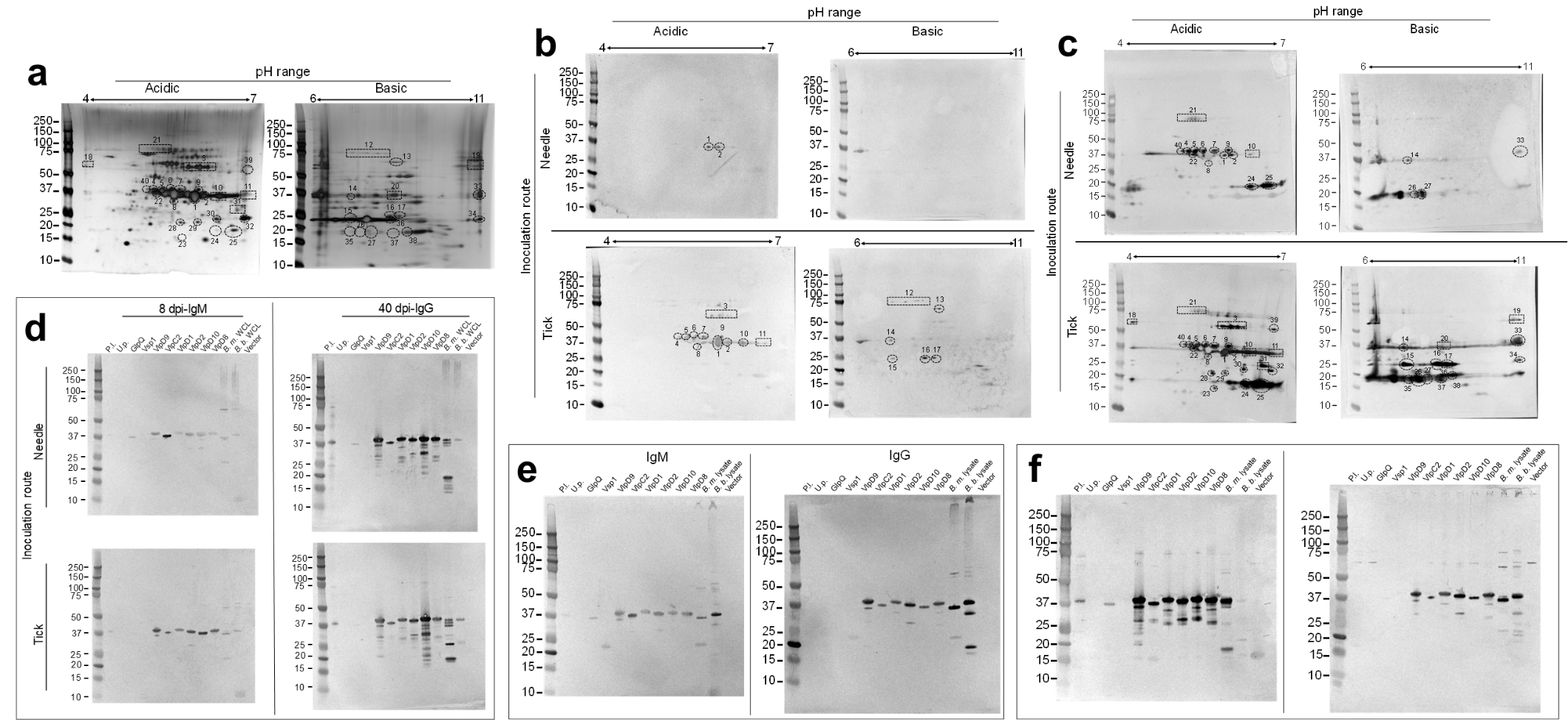


Supplemental Figure 2. Full-length gel and immunoblot images. **a)** Silver stain representation of *B. miyamotoi* LB-2001 membrane-associated proteins from Figure 1. **b)** IgM 2DE immunoblot with sera from *B. miyamotoi*-infected CD1 mice collected at 8 dpi from Figure 2. Antigen recognition was examined in response to needle inoculation of *B. miyamotoi* LB-2001 (top panel) or through tick bite from *B. miyamotoi*-*i*nfected *I. scapularis* originating from Minnesota (bottom panel). For both sets of immunoblots an acidic (pH 4-7) and basic (6-11) pH range were utilized. Sera were diluted 1:200 for immunoblotting. Immunogenic proteins spots (number 1-17) were excised from a corresponding silver stain 2DE and identified by mass spectrometry. **c)** IgG 2DE immunoblot with sera from *B. miyamotoi*-infected CD1 mice collected at 40 dpi from Figure 3. Antigen recognition was examined in response to needle inoculation of *B. miyamotoi* LB-2001 (top panel) or through tick bite from *B. miyamotoi*-infected *I. scapularis* originating from Minnesota (bottom panel). For both sets of immunoblots an acidic (pH 4-7) and basic (6-11) pH range were utilized. Sera were diluted 1:200 for immunoblotting. Immunogenic protein spots (numbers 1-11 and 14-40) were excised from a corresponding silver stain 2DE and identified by mass spectrometry. **d)** Validation of anti-*B. miyamotoi* murine antibodies against recombinant *B. miyamotoi* proteins (100 ng) from Figure 5. IgM response in CD1 mouse serum collected at 8 dpi to needle (top, left panel) or tick (bottom, left panel) inoculation. IgG response in CD1 mouse serum collected at 40 dpi to needle (top, right panel) or tick (bottom, right panel) inoculation. All sera were diluted 1:200 for immunoblotting. **e)** Evaluation of cross-reactive antibodies present in *B. burgdorferi*-infected CD1 mice against recombinant *B. miyamotoi* proteins (100 ng) from Figure 6. Pooled sera from CD1 mice exposed to *B. burgdorferi* (B31)-infected *I. scapularis* was assayed against *B. miyamotoi* recombinant proteins and an IgM (left panel) or IgG (right panel) antibody response observed. Serum was diluted 1:200 for immunoblotting. **f)** Evaluation of IgG reactivity to *B. miyamotoi* recombinant antigens (100 ng) using serum collected from a BMD patient (left panel), and a LD patient (right panel) from Figure 7. Patient serum samples diluted 1:200.
